# Supplementary figures and images for: A new and very spiny lizard (Gymnophthalmidae: Echinosaura) from the Andes in northwestern Ecuador (part 1 of 3)
Source: PeerJ. 2021 Dec 10;9:e12523. doi: 10.7717/peerj.12523 (PMC8667736; doi:10.7717/peerj.12523)

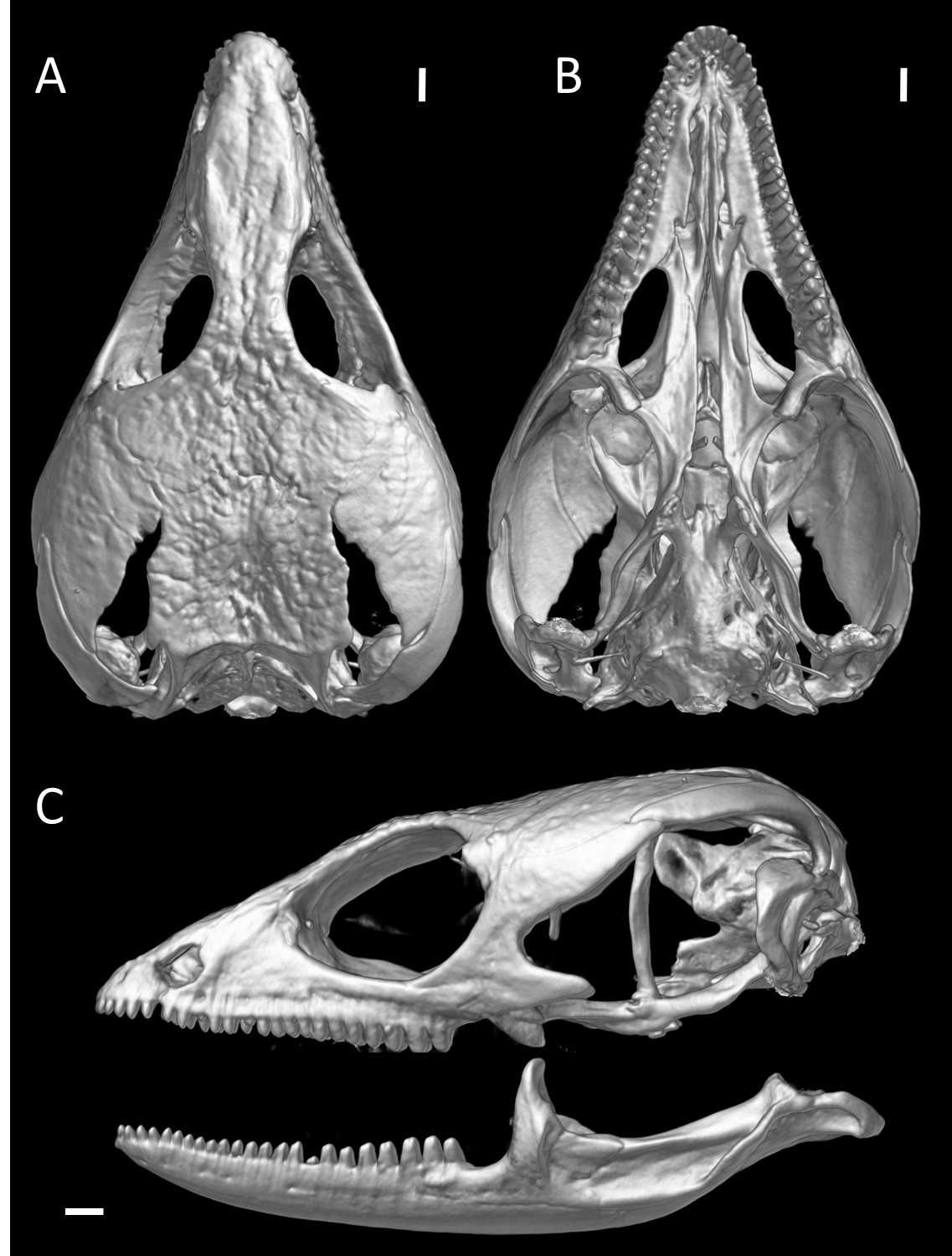

Supplement: Supplemental Information 1 — Scale bar = 1 mm. [file peerj-09-12523-s001.png]

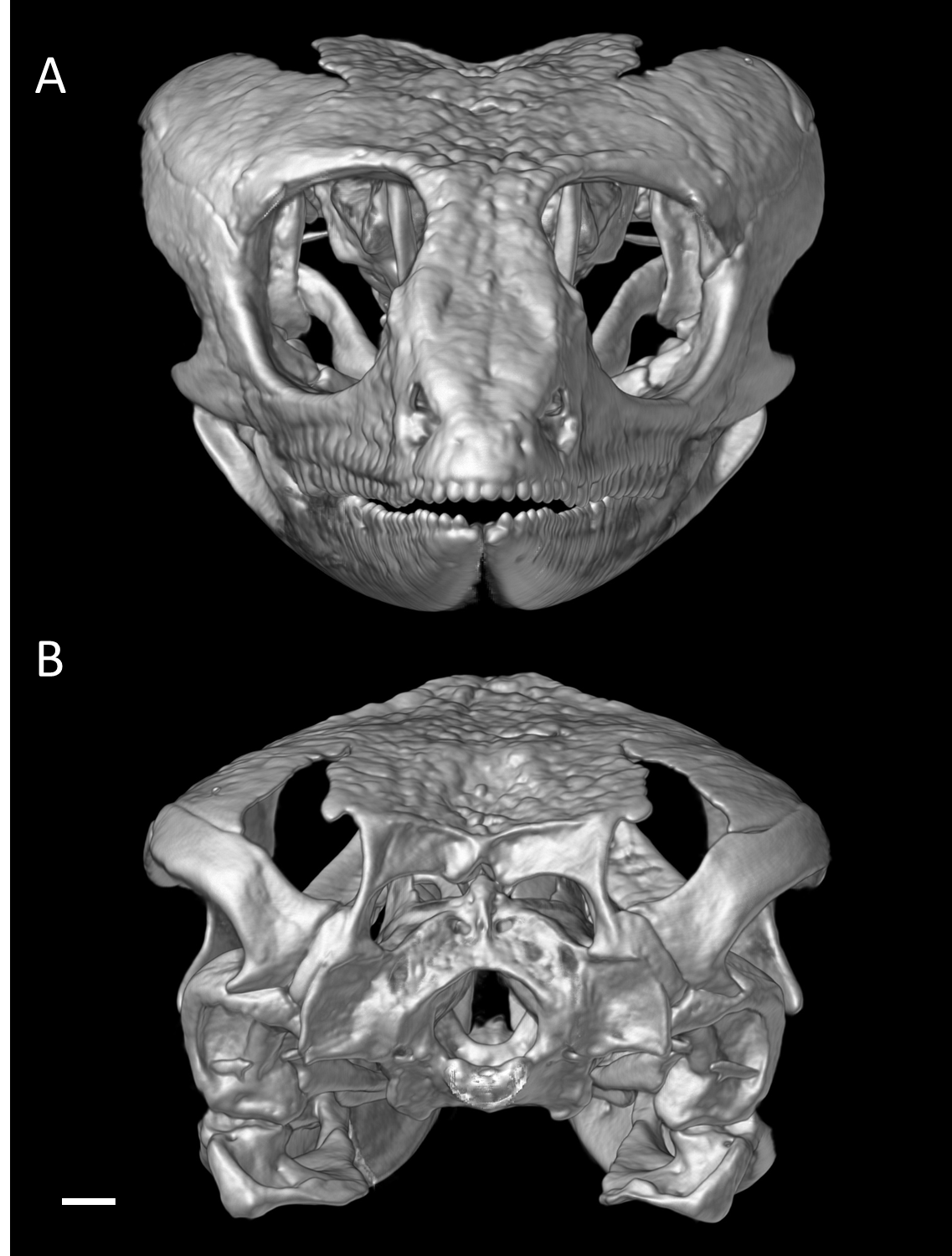

Supplement: Supplemental Information 2 — Scale bar = 1 mm. [file peerj-09-12523-s002.png]

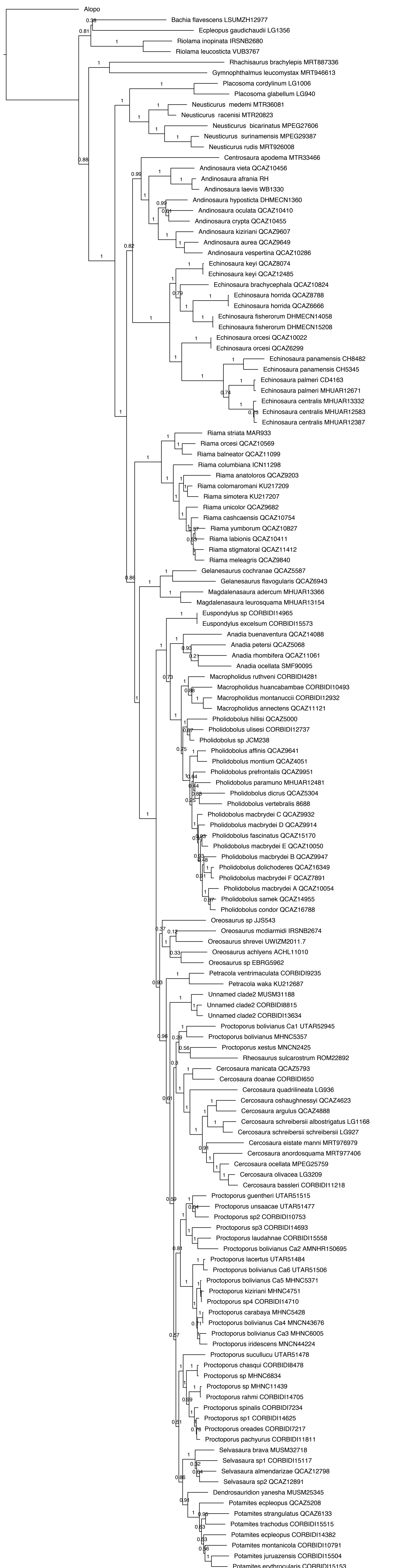

Supplement: Supplemental Information 3 — Maximum clade credibility tree obtained under a Bayesian analysis of 148 taxa and 1,935 characters aligned base pairs of mitochondrial and nuclear DNA. Posterior probabilities are indicated next to branches. [file peerj-09-12523-s003.pdf]

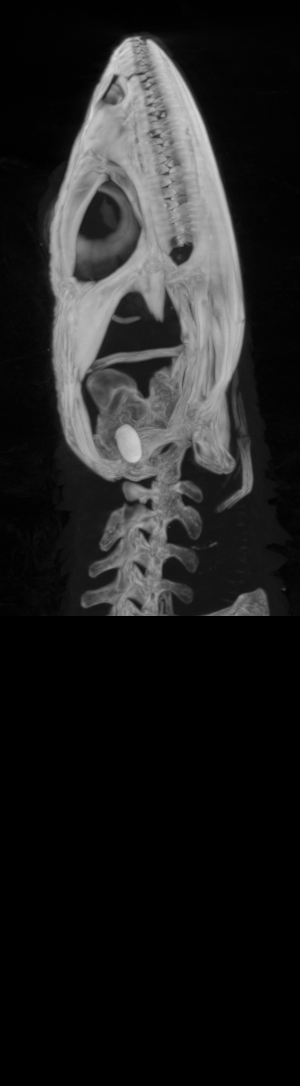

Supplement: Supplemental Information 4 [file peerj-09-12523-s004.zip › Skull_VOI/DHMECN_SC_058_rec_Tra_spr.png]

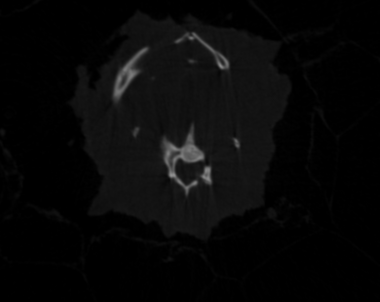

Supplement: Supplemental Information 4 [file peerj-09-12523-s004.zip › Skull_VOI/DHMECN_SC_058_rec_Tra0470.png]

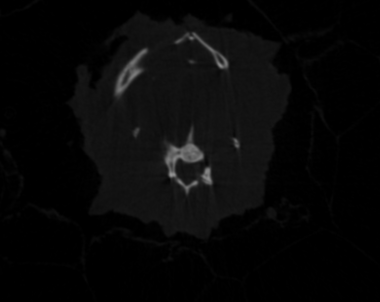

Supplement: Supplemental Information 4 [file peerj-09-12523-s004.zip › Skull_VOI/DHMECN_SC_058_rec_Tra0471.png]

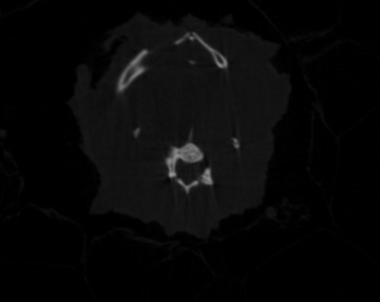

Supplement: Supplemental Information 4 [file peerj-09-12523-s004.zip › Skull_VOI/DHMECN_SC_058_rec_Tra0472.png]

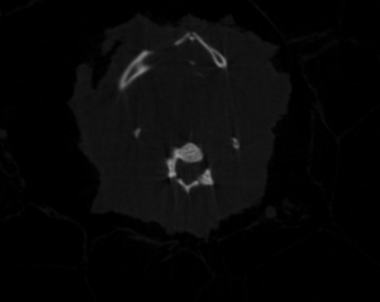

Supplement: Supplemental Information 4 [file peerj-09-12523-s004.zip › Skull_VOI/DHMECN_SC_058_rec_Tra0473.png]

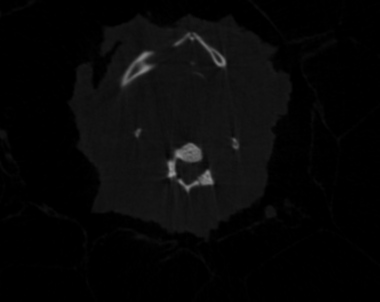

Supplement: Supplemental Information 4 [file peerj-09-12523-s004.zip › Skull_VOI/DHMECN_SC_058_rec_Tra0474.png]

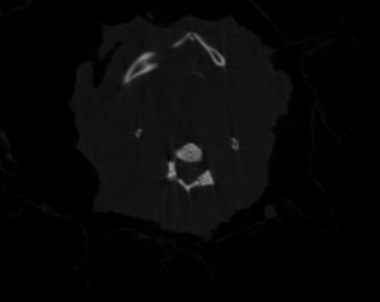

Supplement: Supplemental Information 4 [file peerj-09-12523-s004.zip › Skull_VOI/DHMECN_SC_058_rec_Tra0475.png]

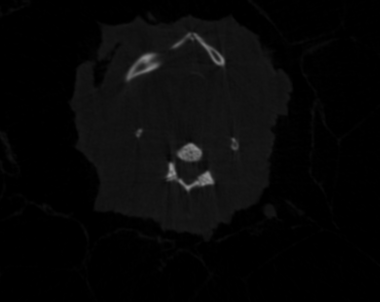

Supplement: Supplemental Information 4 [file peerj-09-12523-s004.zip › Skull_VOI/DHMECN_SC_058_rec_Tra0476.png]

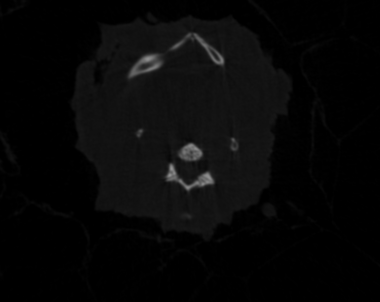

Supplement: Supplemental Information 4 [file peerj-09-12523-s004.zip › Skull_VOI/DHMECN_SC_058_rec_Tra0477.png]

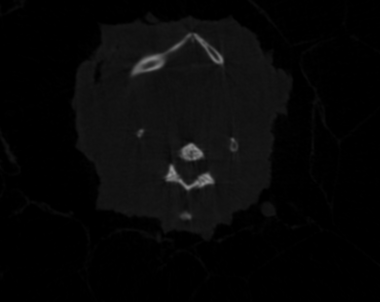

Supplement: Supplemental Information 4 [file peerj-09-12523-s004.zip › Skull_VOI/DHMECN_SC_058_rec_Tra0478.png]

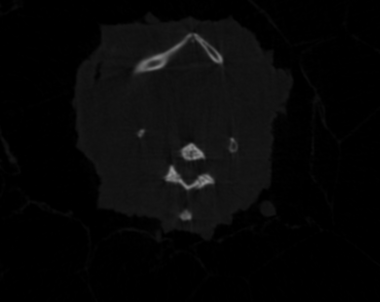

Supplement: Supplemental Information 4 [file peerj-09-12523-s004.zip › Skull_VOI/DHMECN_SC_058_rec_Tra0479.png]

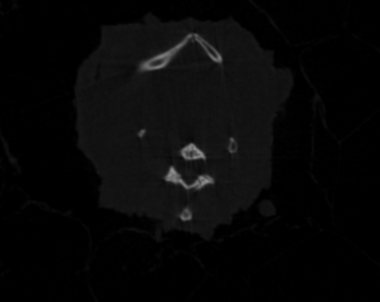

Supplement: Supplemental Information 4 [file peerj-09-12523-s004.zip › Skull_VOI/DHMECN_SC_058_rec_Tra0480.png]

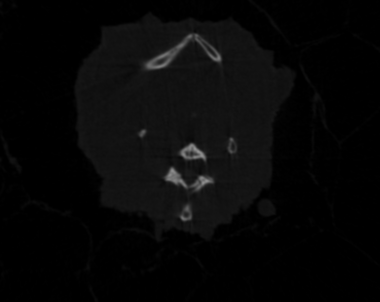

Supplement: Supplemental Information 4 [file peerj-09-12523-s004.zip › Skull_VOI/DHMECN_SC_058_rec_Tra0481.png]

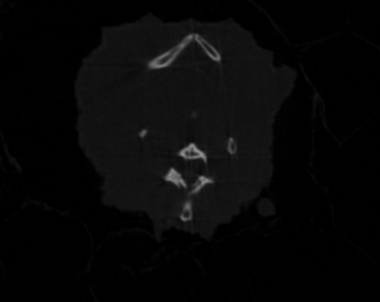

Supplement: Supplemental Information 4 [file peerj-09-12523-s004.zip › Skull_VOI/DHMECN_SC_058_rec_Tra0482.png]

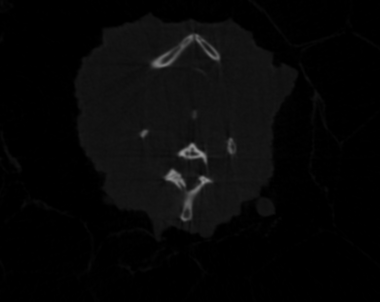

Supplement: Supplemental Information 4 [file peerj-09-12523-s004.zip › Skull_VOI/DHMECN_SC_058_rec_Tra0483.png]

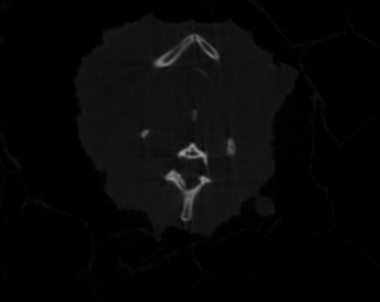

Supplement: Supplemental Information 4 [file peerj-09-12523-s004.zip › Skull_VOI/DHMECN_SC_058_rec_Tra0484.png]

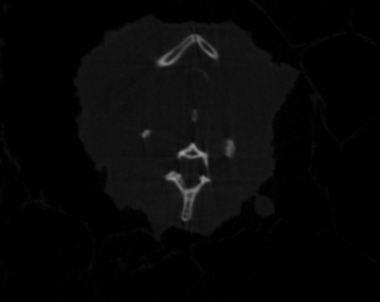

Supplement: Supplemental Information 4 [file peerj-09-12523-s004.zip › Skull_VOI/DHMECN_SC_058_rec_Tra0485.png]

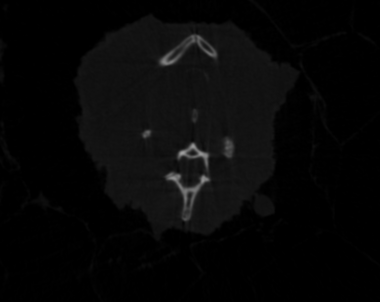

Supplement: Supplemental Information 4 [file peerj-09-12523-s004.zip › Skull_VOI/DHMECN_SC_058_rec_Tra0486.png]

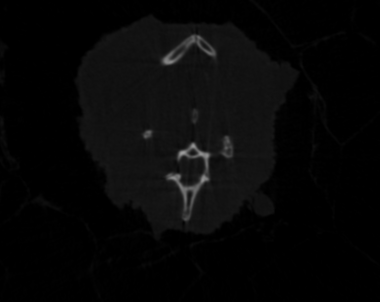

Supplement: Supplemental Information 4 [file peerj-09-12523-s004.zip › Skull_VOI/DHMECN_SC_058_rec_Tra0487.png]

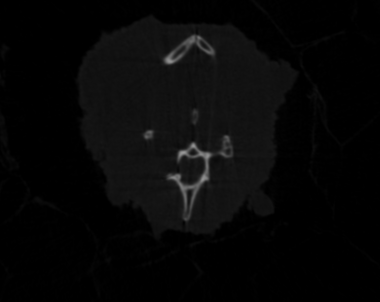

Supplement: Supplemental Information 4 [file peerj-09-12523-s004.zip › Skull_VOI/DHMECN_SC_058_rec_Tra0488.png]

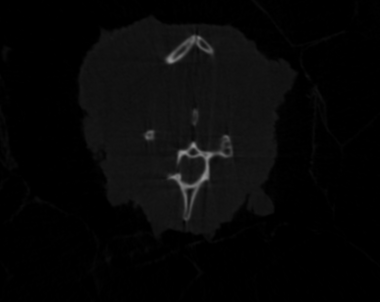

Supplement: Supplemental Information 4 [file peerj-09-12523-s004.zip › Skull_VOI/DHMECN_SC_058_rec_Tra0489.png]

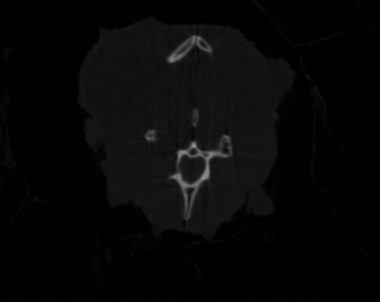

Supplement: Supplemental Information 4 [file peerj-09-12523-s004.zip › Skull_VOI/DHMECN_SC_058_rec_Tra0490.png]

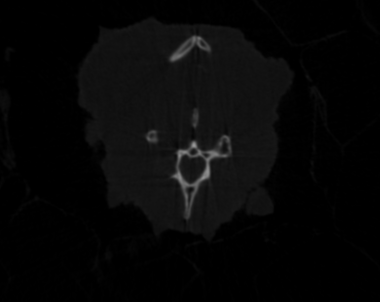

Supplement: Supplemental Information 4 [file peerj-09-12523-s004.zip › Skull_VOI/DHMECN_SC_058_rec_Tra0491.png]

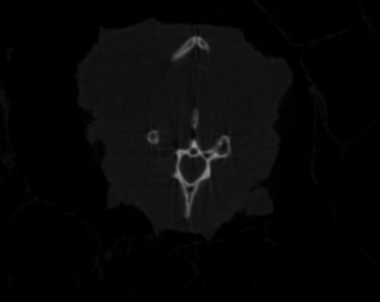

Supplement: Supplemental Information 4 [file peerj-09-12523-s004.zip › Skull_VOI/DHMECN_SC_058_rec_Tra0492.png]

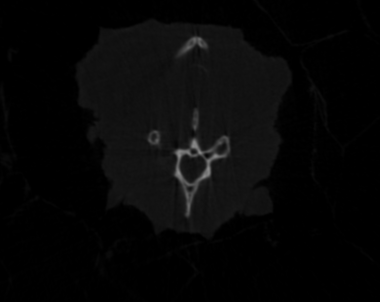

Supplement: Supplemental Information 4 [file peerj-09-12523-s004.zip › Skull_VOI/DHMECN_SC_058_rec_Tra0493.png]

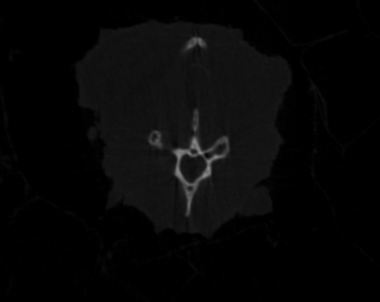

Supplement: Supplemental Information 4 [file peerj-09-12523-s004.zip › Skull_VOI/DHMECN_SC_058_rec_Tra0494.png]

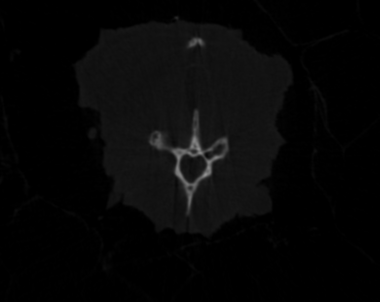

Supplement: Supplemental Information 4 [file peerj-09-12523-s004.zip › Skull_VOI/DHMECN_SC_058_rec_Tra0495.png]

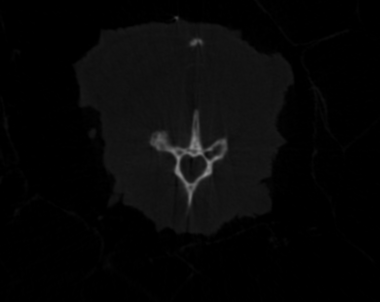

Supplement: Supplemental Information 4 [file peerj-09-12523-s004.zip › Skull_VOI/DHMECN_SC_058_rec_Tra0496.png]

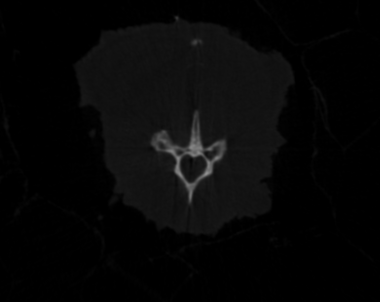

Supplement: Supplemental Information 4 [file peerj-09-12523-s004.zip › Skull_VOI/DHMECN_SC_058_rec_Tra0497.png]

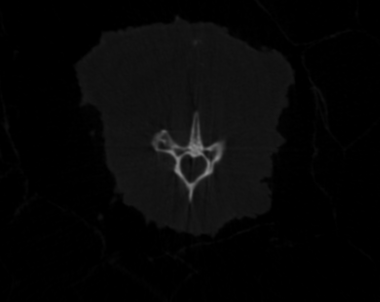

Supplement: Supplemental Information 4 [file peerj-09-12523-s004.zip › Skull_VOI/DHMECN_SC_058_rec_Tra0498.png]

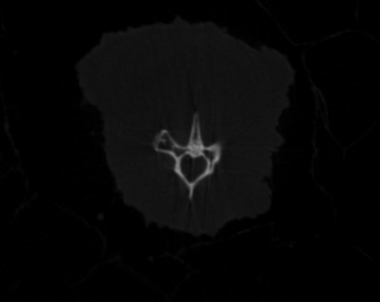

Supplement: Supplemental Information 4 [file peerj-09-12523-s004.zip › Skull_VOI/DHMECN_SC_058_rec_Tra0499.png]

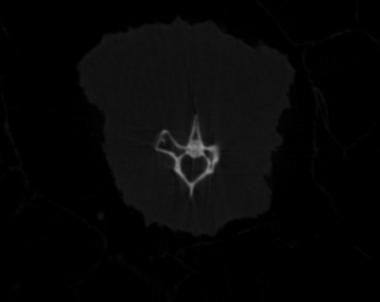

Supplement: Supplemental Information 4 [file peerj-09-12523-s004.zip › Skull_VOI/DHMECN_SC_058_rec_Tra0500.png]

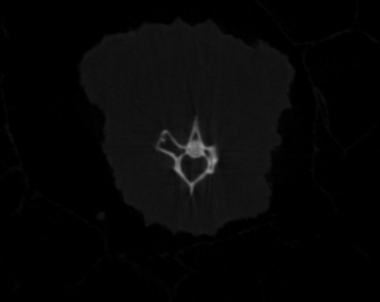

Supplement: Supplemental Information 4 [file peerj-09-12523-s004.zip › Skull_VOI/DHMECN_SC_058_rec_Tra0501.png]

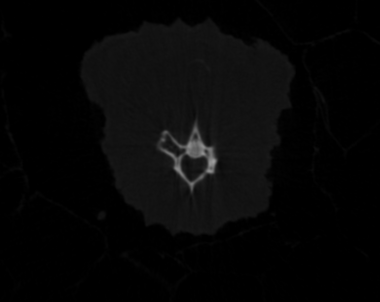

Supplement: Supplemental Information 4 [file peerj-09-12523-s004.zip › Skull_VOI/DHMECN_SC_058_rec_Tra0502.png]

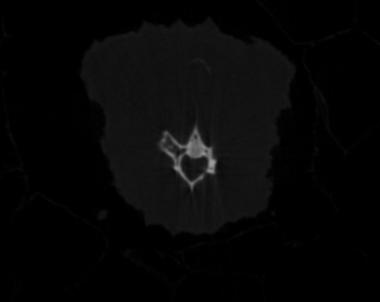

Supplement: Supplemental Information 4 [file peerj-09-12523-s004.zip › Skull_VOI/DHMECN_SC_058_rec_Tra0503.png]

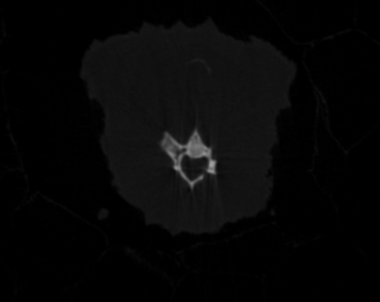

Supplement: Supplemental Information 4 [file peerj-09-12523-s004.zip › Skull_VOI/DHMECN_SC_058_rec_Tra0504.png]

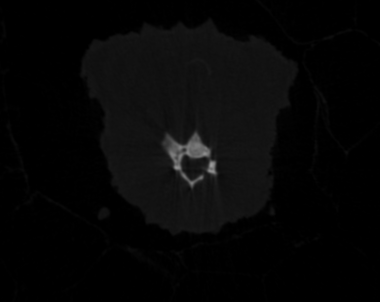

Supplement: Supplemental Information 4 [file peerj-09-12523-s004.zip › Skull_VOI/DHMECN_SC_058_rec_Tra0505.png]

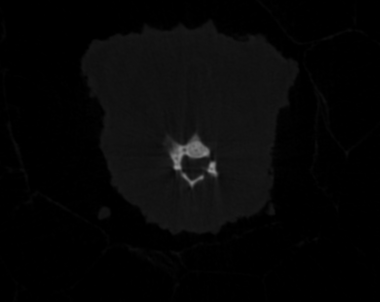

Supplement: Supplemental Information 4 [file peerj-09-12523-s004.zip › Skull_VOI/DHMECN_SC_058_rec_Tra0506.png]

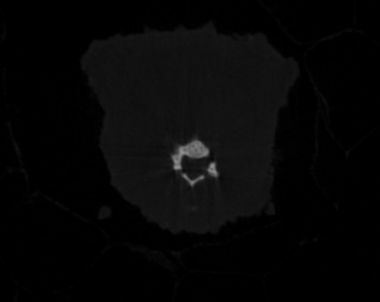

Supplement: Supplemental Information 4 [file peerj-09-12523-s004.zip › Skull_VOI/DHMECN_SC_058_rec_Tra0507.png]

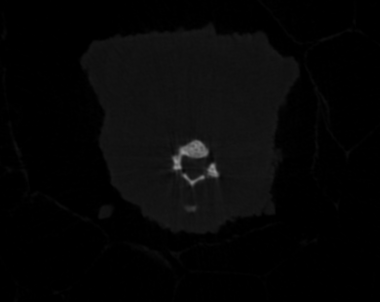

Supplement: Supplemental Information 4 [file peerj-09-12523-s004.zip › Skull_VOI/DHMECN_SC_058_rec_Tra0508.png]

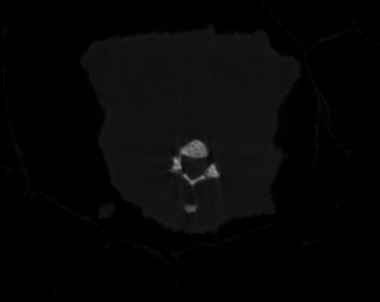

Supplement: Supplemental Information 4 [file peerj-09-12523-s004.zip › Skull_VOI/DHMECN_SC_058_rec_Tra0509.png]

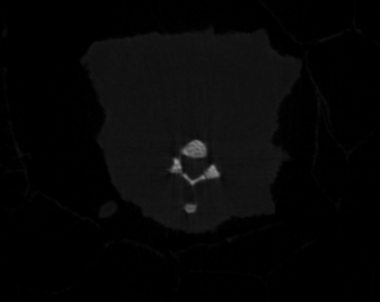

Supplement: Supplemental Information 4 [file peerj-09-12523-s004.zip › Skull_VOI/DHMECN_SC_058_rec_Tra0510.png]

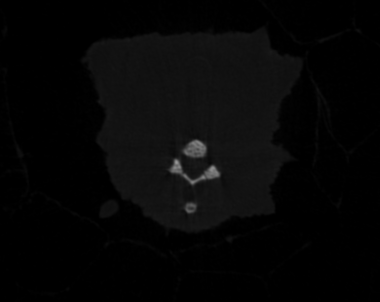

Supplement: Supplemental Information 4 [file peerj-09-12523-s004.zip › Skull_VOI/DHMECN_SC_058_rec_Tra0511.png]

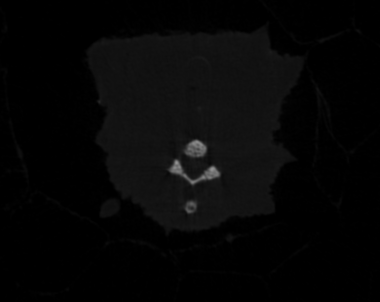

Supplement: Supplemental Information 4 [file peerj-09-12523-s004.zip › Skull_VOI/DHMECN_SC_058_rec_Tra0512.png]

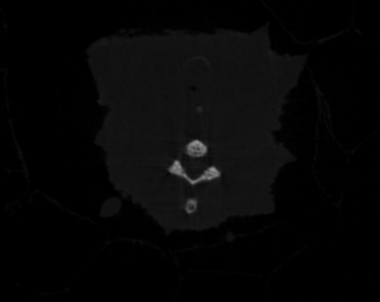

Supplement: Supplemental Information 4 [file peerj-09-12523-s004.zip › Skull_VOI/DHMECN_SC_058_rec_Tra0513.png]

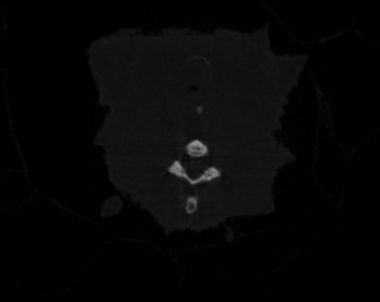

Supplement: Supplemental Information 4 [file peerj-09-12523-s004.zip › Skull_VOI/DHMECN_SC_058_rec_Tra0514.png]

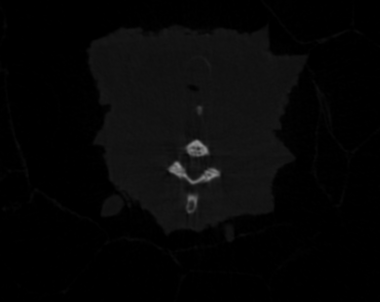

Supplement: Supplemental Information 4 [file peerj-09-12523-s004.zip › Skull_VOI/DHMECN_SC_058_rec_Tra0515.png]

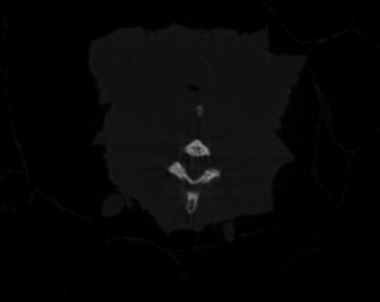

Supplement: Supplemental Information 4 [file peerj-09-12523-s004.zip › Skull_VOI/DHMECN_SC_058_rec_Tra0516.png]

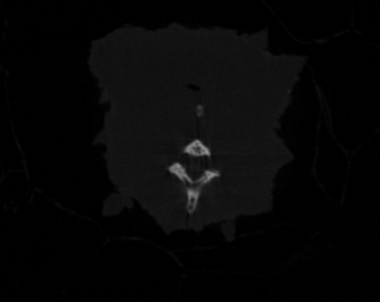

Supplement: Supplemental Information 4 [file peerj-09-12523-s004.zip › Skull_VOI/DHMECN_SC_058_rec_Tra0517.png]

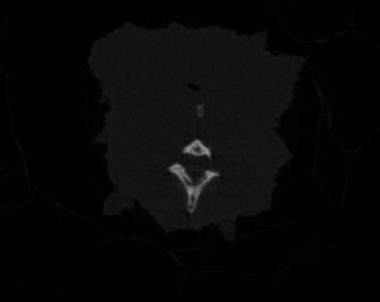

Supplement: Supplemental Information 4 [file peerj-09-12523-s004.zip › Skull_VOI/DHMECN_SC_058_rec_Tra0518.png]

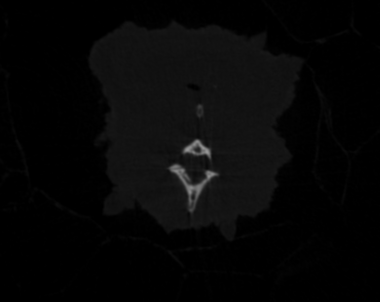

Supplement: Supplemental Information 4 [file peerj-09-12523-s004.zip › Skull_VOI/DHMECN_SC_058_rec_Tra0519.png]

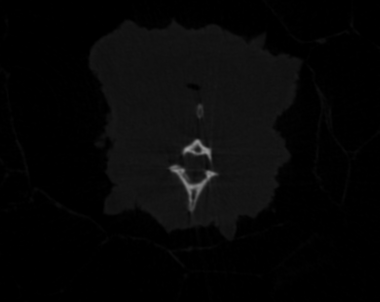

Supplement: Supplemental Information 4 [file peerj-09-12523-s004.zip › Skull_VOI/DHMECN_SC_058_rec_Tra0520.png]

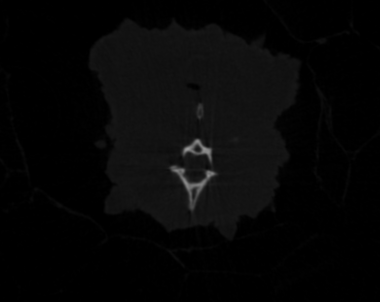

Supplement: Supplemental Information 4 [file peerj-09-12523-s004.zip › Skull_VOI/DHMECN_SC_058_rec_Tra0521.png]

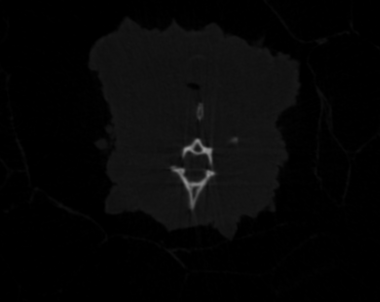

Supplement: Supplemental Information 4 [file peerj-09-12523-s004.zip › Skull_VOI/DHMECN_SC_058_rec_Tra0522.png]

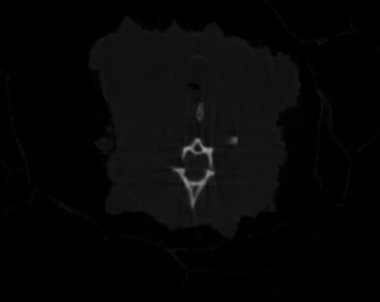

Supplement: Supplemental Information 4 [file peerj-09-12523-s004.zip › Skull_VOI/DHMECN_SC_058_rec_Tra0523.png]

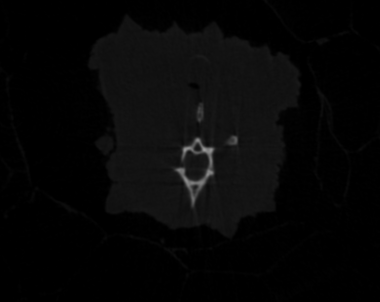

Supplement: Supplemental Information 4 [file peerj-09-12523-s004.zip › Skull_VOI/DHMECN_SC_058_rec_Tra0524.png]

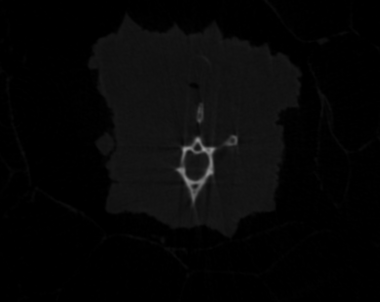

Supplement: Supplemental Information 4 [file peerj-09-12523-s004.zip › Skull_VOI/DHMECN_SC_058_rec_Tra0525.png]

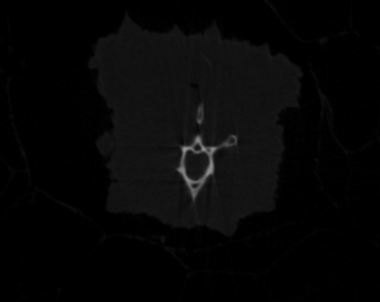

Supplement: Supplemental Information 4 [file peerj-09-12523-s004.zip › Skull_VOI/DHMECN_SC_058_rec_Tra0526.png]

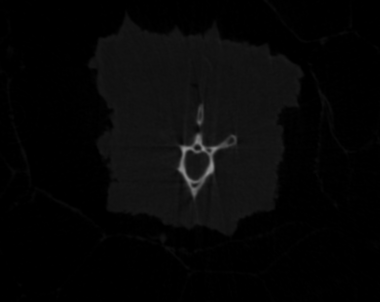

Supplement: Supplemental Information 4 [file peerj-09-12523-s004.zip › Skull_VOI/DHMECN_SC_058_rec_Tra0527.png]

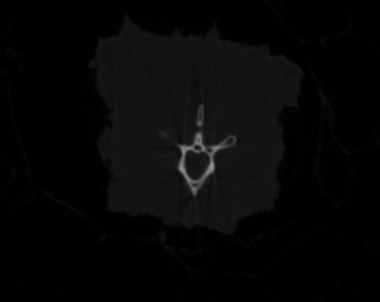

Supplement: Supplemental Information 4 [file peerj-09-12523-s004.zip › Skull_VOI/DHMECN_SC_058_rec_Tra0528.png]

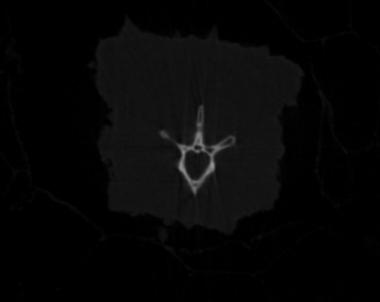

Supplement: Supplemental Information 4 [file peerj-09-12523-s004.zip › Skull_VOI/DHMECN_SC_058_rec_Tra0529.png]

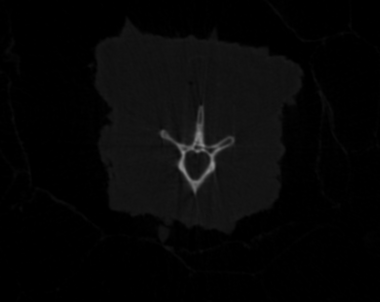

Supplement: Supplemental Information 4 [file peerj-09-12523-s004.zip › Skull_VOI/DHMECN_SC_058_rec_Tra0530.png]

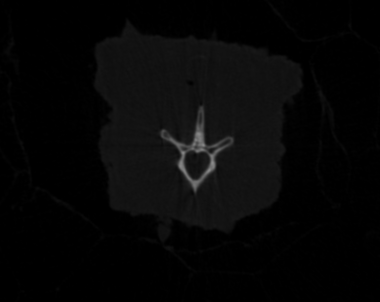

Supplement: Supplemental Information 4 [file peerj-09-12523-s004.zip › Skull_VOI/DHMECN_SC_058_rec_Tra0531.png]

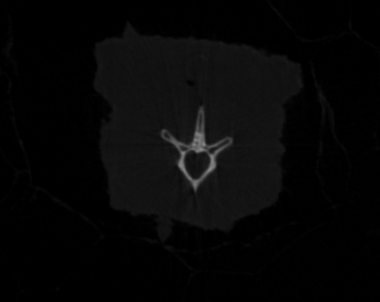

Supplement: Supplemental Information 4 [file peerj-09-12523-s004.zip › Skull_VOI/DHMECN_SC_058_rec_Tra0532.png]

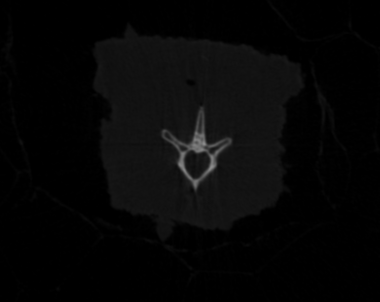

Supplement: Supplemental Information 4 [file peerj-09-12523-s004.zip › Skull_VOI/DHMECN_SC_058_rec_Tra0533.png]

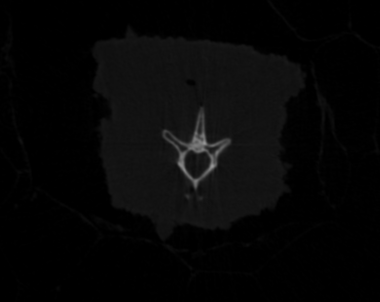

Supplement: Supplemental Information 4 [file peerj-09-12523-s004.zip › Skull_VOI/DHMECN_SC_058_rec_Tra0534.png]

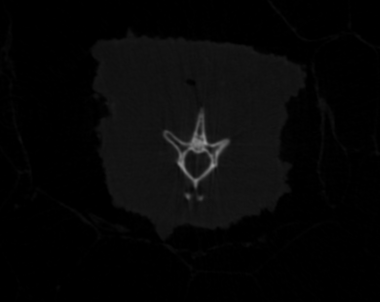

Supplement: Supplemental Information 4 [file peerj-09-12523-s004.zip › Skull_VOI/DHMECN_SC_058_rec_Tra0535.png]

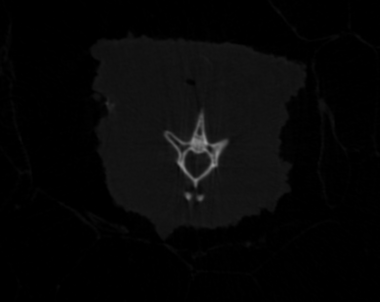

Supplement: Supplemental Information 4 [file peerj-09-12523-s004.zip › Skull_VOI/DHMECN_SC_058_rec_Tra0536.png]

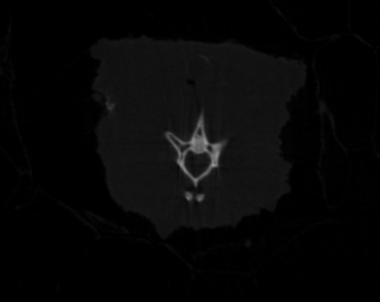

Supplement: Supplemental Information 4 [file peerj-09-12523-s004.zip › Skull_VOI/DHMECN_SC_058_rec_Tra0537.png]

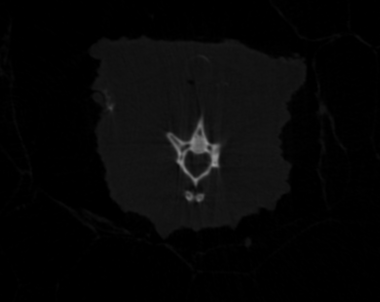

Supplement: Supplemental Information 4 [file peerj-09-12523-s004.zip › Skull_VOI/DHMECN_SC_058_rec_Tra0538.png]

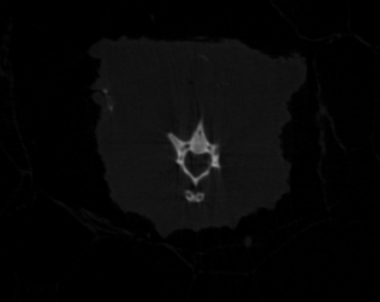

Supplement: Supplemental Information 4 [file peerj-09-12523-s004.zip › Skull_VOI/DHMECN_SC_058_rec_Tra0539.png]

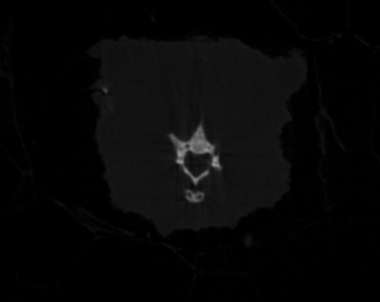

Supplement: Supplemental Information 4 [file peerj-09-12523-s004.zip › Skull_VOI/DHMECN_SC_058_rec_Tra0540.png]

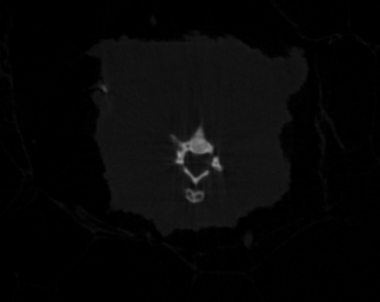

Supplement: Supplemental Information 4 [file peerj-09-12523-s004.zip › Skull_VOI/DHMECN_SC_058_rec_Tra0541.png]

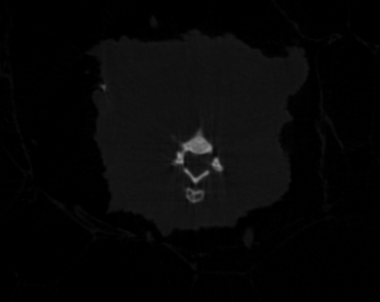

Supplement: Supplemental Information 4 [file peerj-09-12523-s004.zip › Skull_VOI/DHMECN_SC_058_rec_Tra0542.png]

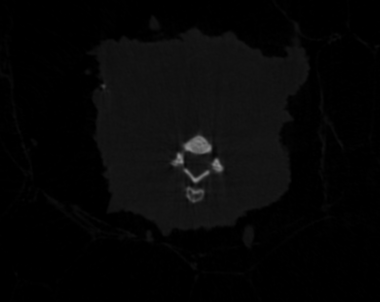

Supplement: Supplemental Information 4 [file peerj-09-12523-s004.zip › Skull_VOI/DHMECN_SC_058_rec_Tra0543.png]

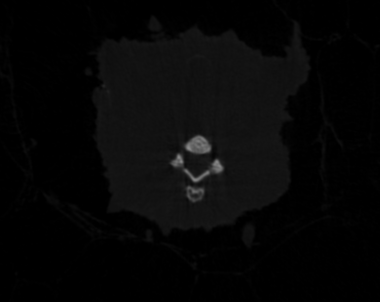

Supplement: Supplemental Information 4 [file peerj-09-12523-s004.zip › Skull_VOI/DHMECN_SC_058_rec_Tra0544.png]

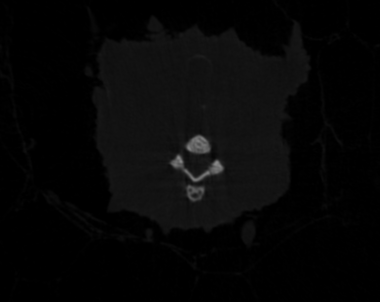

Supplement: Supplemental Information 4 [file peerj-09-12523-s004.zip › Skull_VOI/DHMECN_SC_058_rec_Tra0545.png]

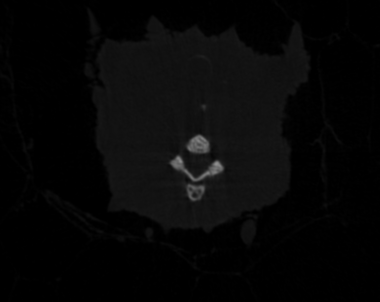

Supplement: Supplemental Information 4 [file peerj-09-12523-s004.zip › Skull_VOI/DHMECN_SC_058_rec_Tra0546.png]

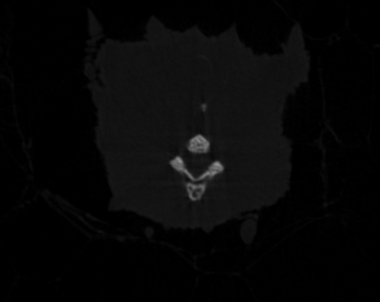

Supplement: Supplemental Information 4 [file peerj-09-12523-s004.zip › Skull_VOI/DHMECN_SC_058_rec_Tra0547.png]

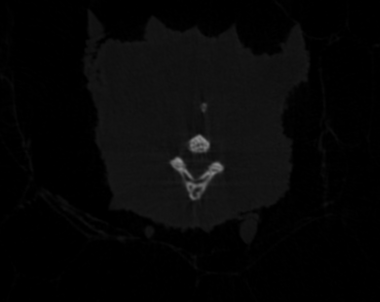

Supplement: Supplemental Information 4 [file peerj-09-12523-s004.zip › Skull_VOI/DHMECN_SC_058_rec_Tra0548.png]

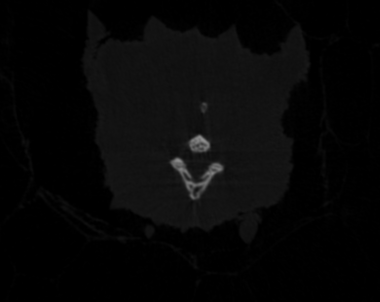

Supplement: Supplemental Information 4 [file peerj-09-12523-s004.zip › Skull_VOI/DHMECN_SC_058_rec_Tra0549.png]

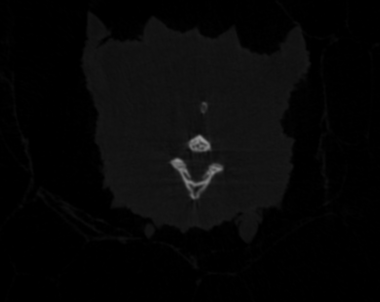

Supplement: Supplemental Information 4 [file peerj-09-12523-s004.zip › Skull_VOI/DHMECN_SC_058_rec_Tra0550.png]

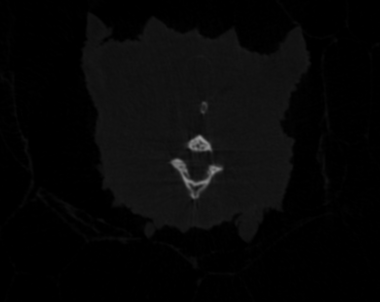

Supplement: Supplemental Information 4 [file peerj-09-12523-s004.zip › Skull_VOI/DHMECN_SC_058_rec_Tra0551.png]

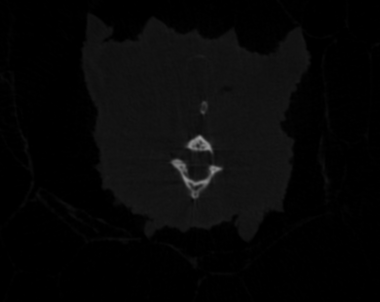

Supplement: Supplemental Information 4 [file peerj-09-12523-s004.zip › Skull_VOI/DHMECN_SC_058_rec_Tra0552.png]

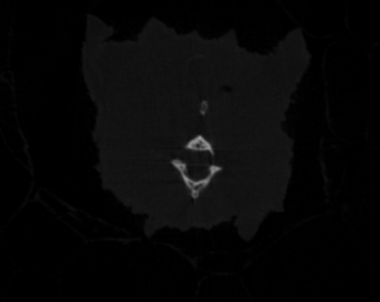

Supplement: Supplemental Information 4 [file peerj-09-12523-s004.zip › Skull_VOI/DHMECN_SC_058_rec_Tra0553.png]

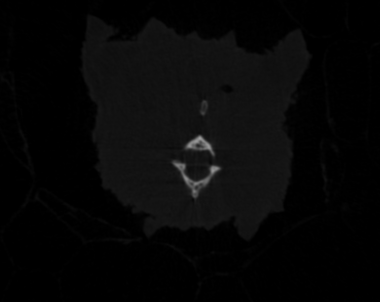

Supplement: Supplemental Information 4 [file peerj-09-12523-s004.zip › Skull_VOI/DHMECN_SC_058_rec_Tra0554.png]

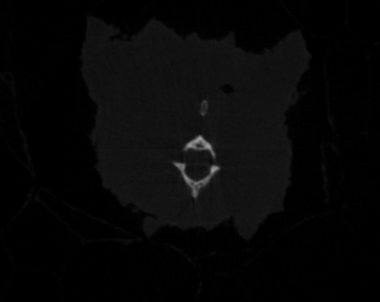

Supplement: Supplemental Information 4 [file peerj-09-12523-s004.zip › Skull_VOI/DHMECN_SC_058_rec_Tra0555.png]

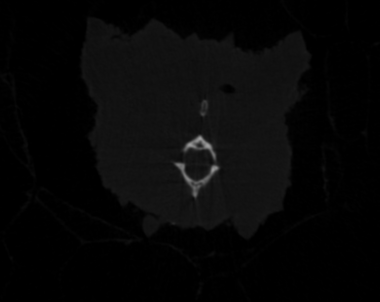

Supplement: Supplemental Information 4 [file peerj-09-12523-s004.zip › Skull_VOI/DHMECN_SC_058_rec_Tra0556.png]

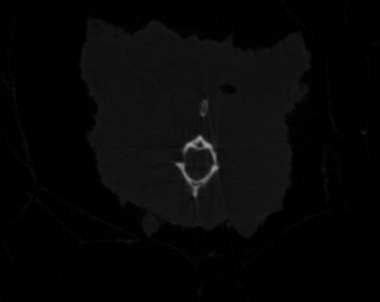

Supplement: Supplemental Information 4 [file peerj-09-12523-s004.zip › Skull_VOI/DHMECN_SC_058_rec_Tra0557.png]

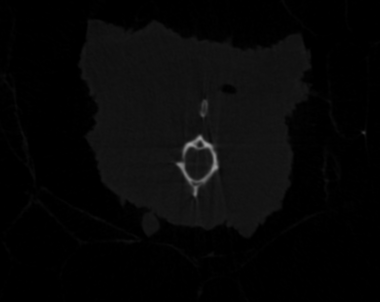

Supplement: Supplemental Information 4 [file peerj-09-12523-s004.zip › Skull_VOI/DHMECN_SC_058_rec_Tra0558.png]

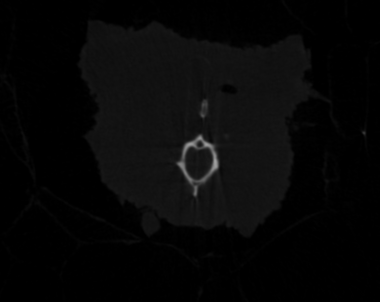

Supplement: Supplemental Information 4 [file peerj-09-12523-s004.zip › Skull_VOI/DHMECN_SC_058_rec_Tra0559.png]

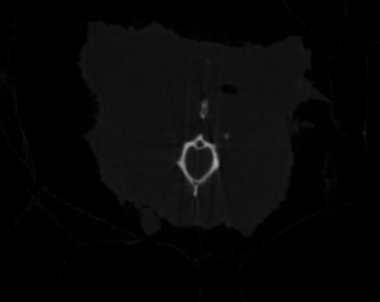

Supplement: Supplemental Information 4 [file peerj-09-12523-s004.zip › Skull_VOI/DHMECN_SC_058_rec_Tra0560.png]

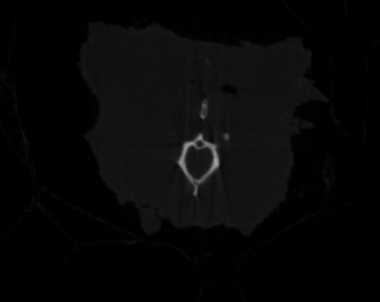

Supplement: Supplemental Information 4 [file peerj-09-12523-s004.zip › Skull_VOI/DHMECN_SC_058_rec_Tra0561.png]

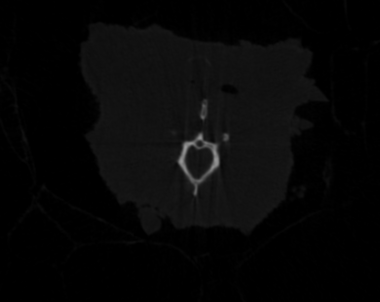

Supplement: Supplemental Information 4 [file peerj-09-12523-s004.zip › Skull_VOI/DHMECN_SC_058_rec_Tra0562.png]

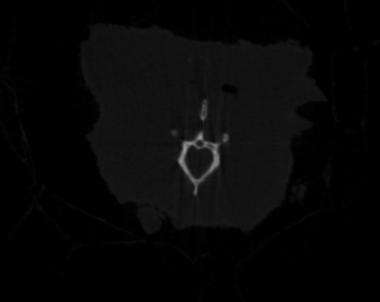

Supplement: Supplemental Information 4 [file peerj-09-12523-s004.zip › Skull_VOI/DHMECN_SC_058_rec_Tra0563.png]

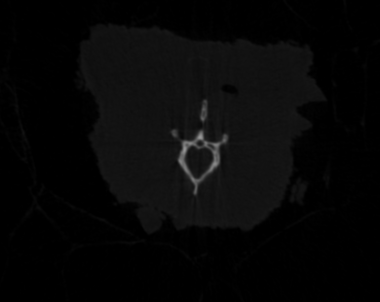

Supplement: Supplemental Information 4 [file peerj-09-12523-s004.zip › Skull_VOI/DHMECN_SC_058_rec_Tra0564.png]

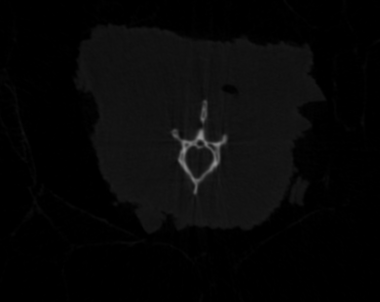

Supplement: Supplemental Information 4 [file peerj-09-12523-s004.zip › Skull_VOI/DHMECN_SC_058_rec_Tra0565.png]
