# Supplementary figures and images for: A new and very spiny lizard (Gymnophthalmidae: Echinosaura) from the Andes in northwestern Ecuador (part 3 of 3)
Source: PeerJ. 2021 Dec 10;9:e12523. doi: 10.7717/peerj.12523 (PMC8667736; doi:10.7717/peerj.12523)

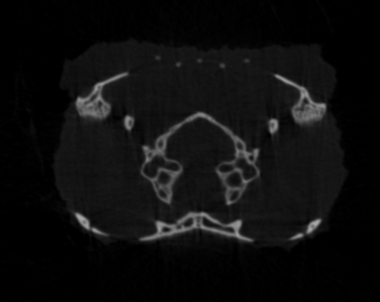

Supplement: Supplemental Information 4 [file peerj-09-12523-s004.zip › Skull_VOI/DHMECN_SC_058_rec_Tra0666.png]

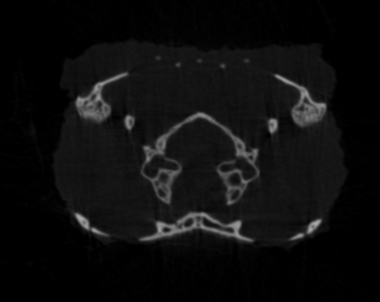

Supplement: Supplemental Information 4 [file peerj-09-12523-s004.zip › Skull_VOI/DHMECN_SC_058_rec_Tra0667.png]
