# Supplementary figures and images for: A new and very spiny lizard (Gymnophthalmidae: Echinosaura) from the Andes in northwestern Ecuador (part 2 of 3)
Source: PeerJ. 2021 Dec 10;9:e12523. doi: 10.7717/peerj.12523 (PMC8667736; doi:10.7717/peerj.12523)

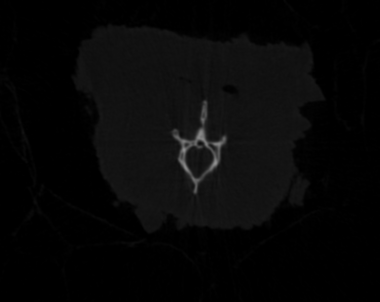

Supplement: Supplemental Information 4 [file peerj-09-12523-s004.zip › Skull_VOI/DHMECN_SC_058_rec_Tra0566.png]

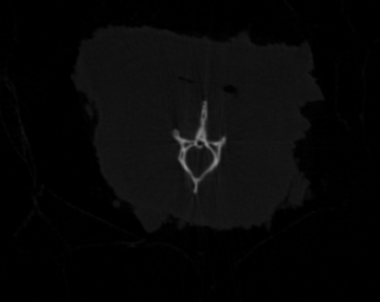

Supplement: Supplemental Information 4 [file peerj-09-12523-s004.zip › Skull_VOI/DHMECN_SC_058_rec_Tra0567.png]

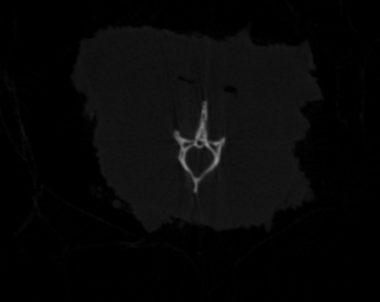

Supplement: Supplemental Information 4 [file peerj-09-12523-s004.zip › Skull_VOI/DHMECN_SC_058_rec_Tra0568.png]

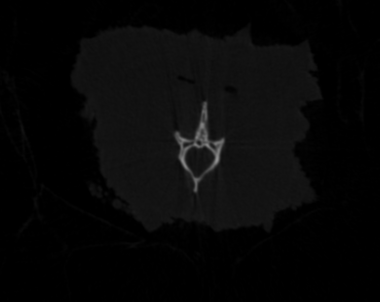

Supplement: Supplemental Information 4 [file peerj-09-12523-s004.zip › Skull_VOI/DHMECN_SC_058_rec_Tra0569.png]

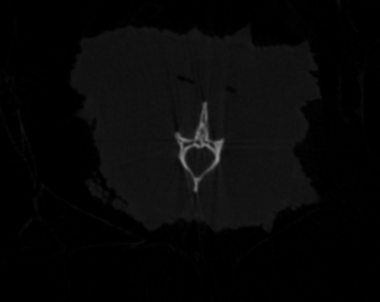

Supplement: Supplemental Information 4 [file peerj-09-12523-s004.zip › Skull_VOI/DHMECN_SC_058_rec_Tra0570.png]

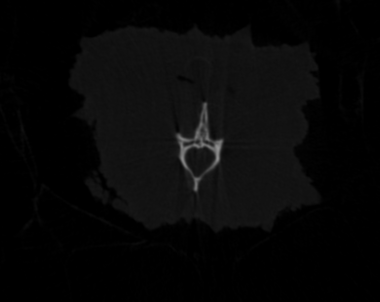

Supplement: Supplemental Information 4 [file peerj-09-12523-s004.zip › Skull_VOI/DHMECN_SC_058_rec_Tra0571.png]

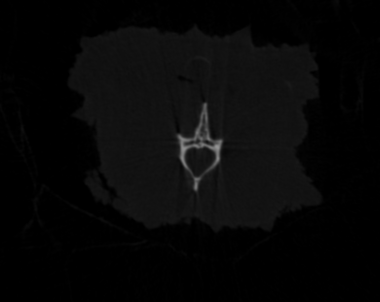

Supplement: Supplemental Information 4 [file peerj-09-12523-s004.zip › Skull_VOI/DHMECN_SC_058_rec_Tra0572.png]

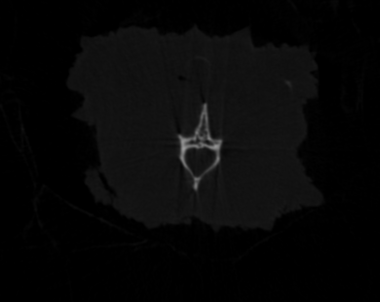

Supplement: Supplemental Information 4 [file peerj-09-12523-s004.zip › Skull_VOI/DHMECN_SC_058_rec_Tra0573.png]

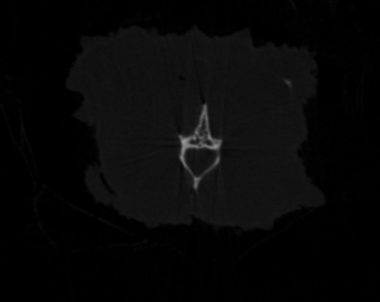

Supplement: Supplemental Information 4 [file peerj-09-12523-s004.zip › Skull_VOI/DHMECN_SC_058_rec_Tra0574.png]

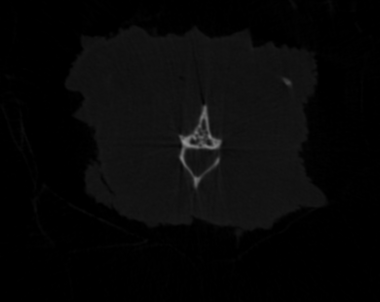

Supplement: Supplemental Information 4 [file peerj-09-12523-s004.zip › Skull_VOI/DHMECN_SC_058_rec_Tra0575.png]

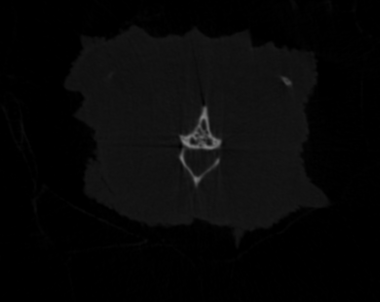

Supplement: Supplemental Information 4 [file peerj-09-12523-s004.zip › Skull_VOI/DHMECN_SC_058_rec_Tra0576.png]

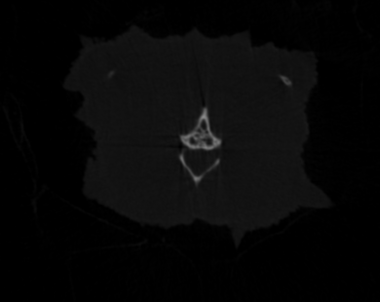

Supplement: Supplemental Information 4 [file peerj-09-12523-s004.zip › Skull_VOI/DHMECN_SC_058_rec_Tra0577.png]

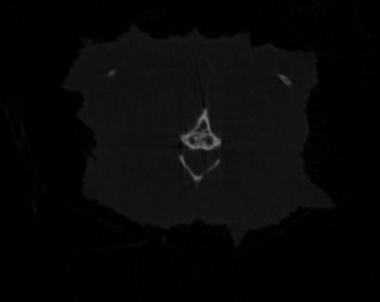

Supplement: Supplemental Information 4 [file peerj-09-12523-s004.zip › Skull_VOI/DHMECN_SC_058_rec_Tra0578.png]

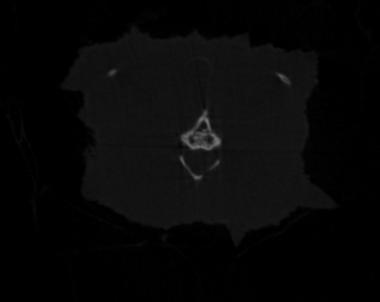

Supplement: Supplemental Information 4 [file peerj-09-12523-s004.zip › Skull_VOI/DHMECN_SC_058_rec_Tra0579.png]

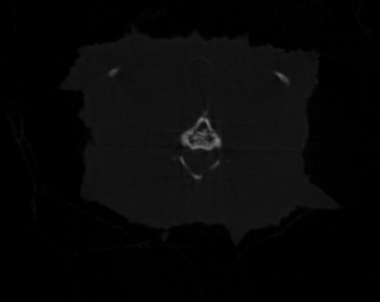

Supplement: Supplemental Information 4 [file peerj-09-12523-s004.zip › Skull_VOI/DHMECN_SC_058_rec_Tra0580.png]

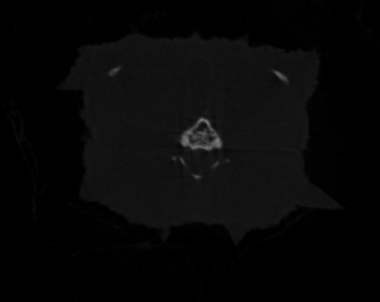

Supplement: Supplemental Information 4 [file peerj-09-12523-s004.zip › Skull_VOI/DHMECN_SC_058_rec_Tra0581.png]

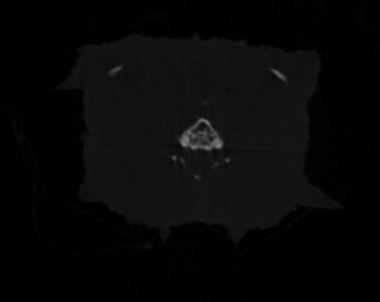

Supplement: Supplemental Information 4 [file peerj-09-12523-s004.zip › Skull_VOI/DHMECN_SC_058_rec_Tra0582.png]

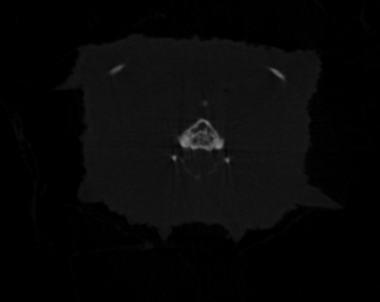

Supplement: Supplemental Information 4 [file peerj-09-12523-s004.zip › Skull_VOI/DHMECN_SC_058_rec_Tra0583.png]

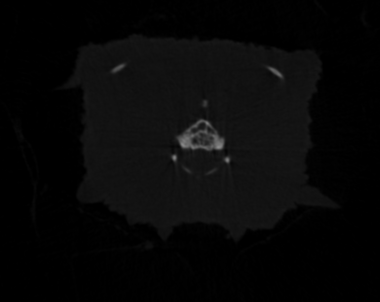

Supplement: Supplemental Information 4 [file peerj-09-12523-s004.zip › Skull_VOI/DHMECN_SC_058_rec_Tra0584.png]

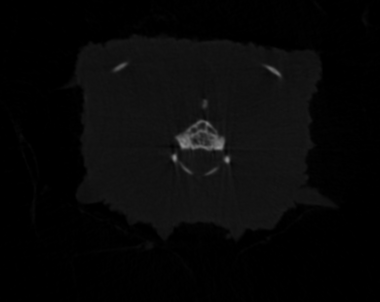

Supplement: Supplemental Information 4 [file peerj-09-12523-s004.zip › Skull_VOI/DHMECN_SC_058_rec_Tra0585.png]

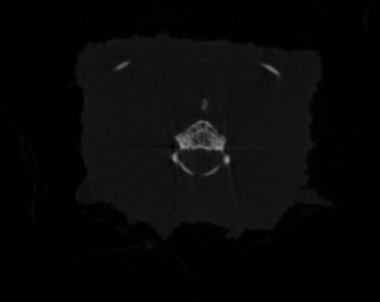

Supplement: Supplemental Information 4 [file peerj-09-12523-s004.zip › Skull_VOI/DHMECN_SC_058_rec_Tra0586.png]

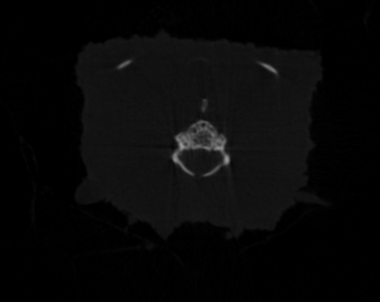

Supplement: Supplemental Information 4 [file peerj-09-12523-s004.zip › Skull_VOI/DHMECN_SC_058_rec_Tra0587.png]

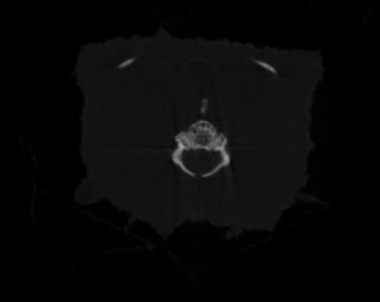

Supplement: Supplemental Information 4 [file peerj-09-12523-s004.zip › Skull_VOI/DHMECN_SC_058_rec_Tra0588.png]

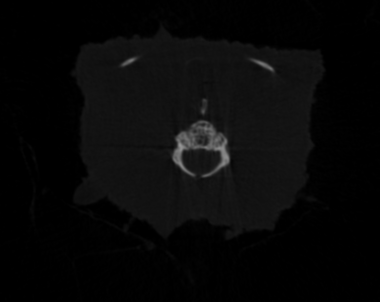

Supplement: Supplemental Information 4 [file peerj-09-12523-s004.zip › Skull_VOI/DHMECN_SC_058_rec_Tra0589.png]

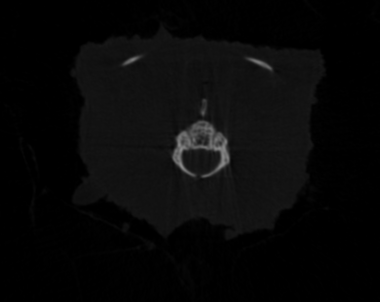

Supplement: Supplemental Information 4 [file peerj-09-12523-s004.zip › Skull_VOI/DHMECN_SC_058_rec_Tra0590.png]

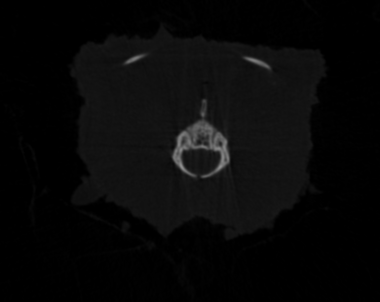

Supplement: Supplemental Information 4 [file peerj-09-12523-s004.zip › Skull_VOI/DHMECN_SC_058_rec_Tra0591.png]

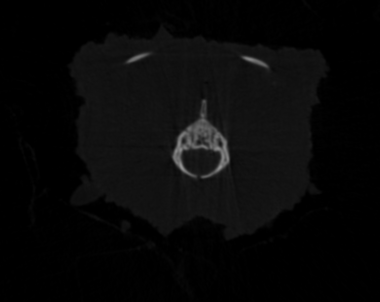

Supplement: Supplemental Information 4 [file peerj-09-12523-s004.zip › Skull_VOI/DHMECN_SC_058_rec_Tra0592.png]

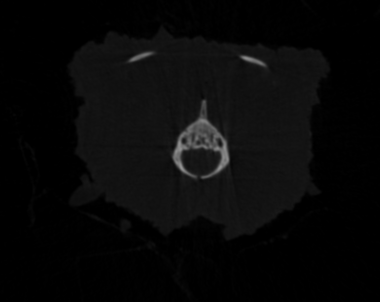

Supplement: Supplemental Information 4 [file peerj-09-12523-s004.zip › Skull_VOI/DHMECN_SC_058_rec_Tra0593.png]

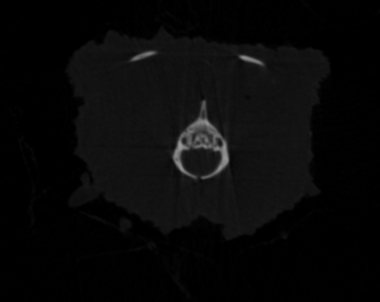

Supplement: Supplemental Information 4 [file peerj-09-12523-s004.zip › Skull_VOI/DHMECN_SC_058_rec_Tra0594.png]

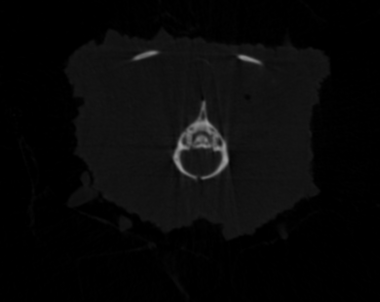

Supplement: Supplemental Information 4 [file peerj-09-12523-s004.zip › Skull_VOI/DHMECN_SC_058_rec_Tra0595.png]

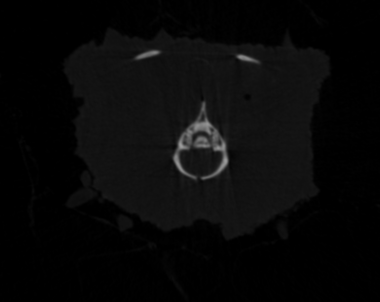

Supplement: Supplemental Information 4 [file peerj-09-12523-s004.zip › Skull_VOI/DHMECN_SC_058_rec_Tra0596.png]

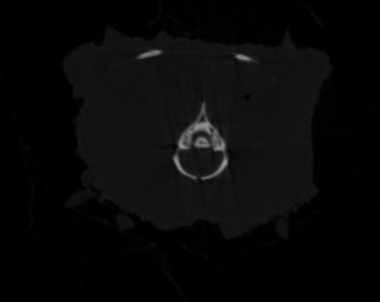

Supplement: Supplemental Information 4 [file peerj-09-12523-s004.zip › Skull_VOI/DHMECN_SC_058_rec_Tra0597.png]

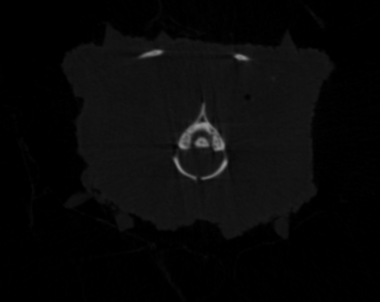

Supplement: Supplemental Information 4 [file peerj-09-12523-s004.zip › Skull_VOI/DHMECN_SC_058_rec_Tra0598.png]

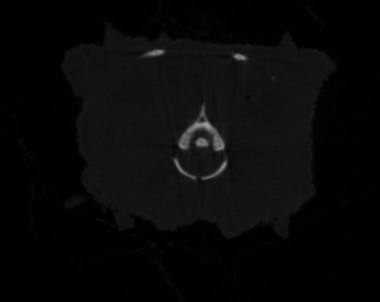

Supplement: Supplemental Information 4 [file peerj-09-12523-s004.zip › Skull_VOI/DHMECN_SC_058_rec_Tra0599.png]

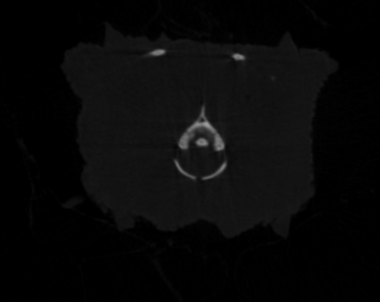

Supplement: Supplemental Information 4 [file peerj-09-12523-s004.zip › Skull_VOI/DHMECN_SC_058_rec_Tra0600.png]

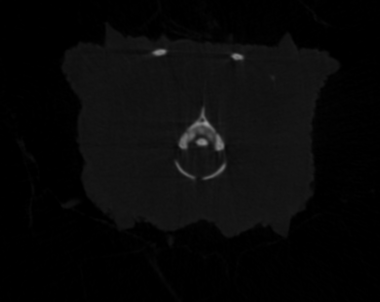

Supplement: Supplemental Information 4 [file peerj-09-12523-s004.zip › Skull_VOI/DHMECN_SC_058_rec_Tra0601.png]

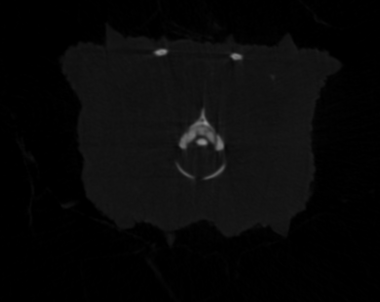

Supplement: Supplemental Information 4 [file peerj-09-12523-s004.zip › Skull_VOI/DHMECN_SC_058_rec_Tra0602.png]

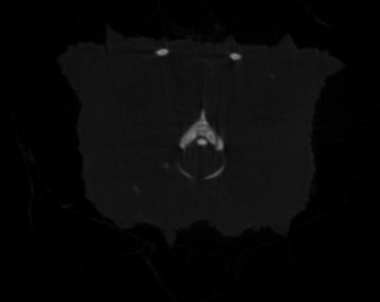

Supplement: Supplemental Information 4 [file peerj-09-12523-s004.zip › Skull_VOI/DHMECN_SC_058_rec_Tra0603.png]

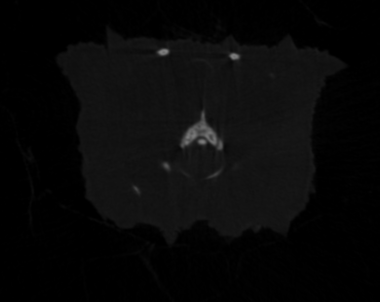

Supplement: Supplemental Information 4 [file peerj-09-12523-s004.zip › Skull_VOI/DHMECN_SC_058_rec_Tra0604.png]

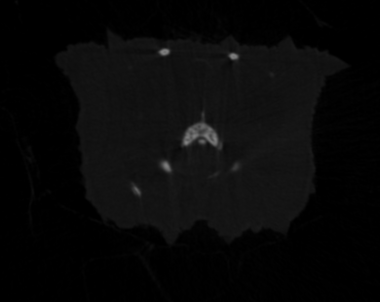

Supplement: Supplemental Information 4 [file peerj-09-12523-s004.zip › Skull_VOI/DHMECN_SC_058_rec_Tra0605.png]

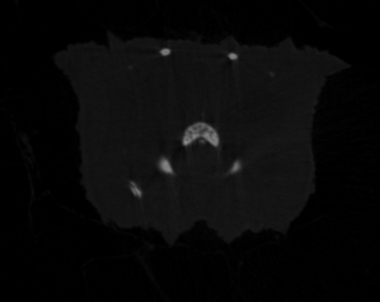

Supplement: Supplemental Information 4 [file peerj-09-12523-s004.zip › Skull_VOI/DHMECN_SC_058_rec_Tra0606.png]

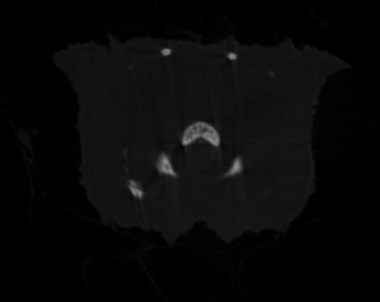

Supplement: Supplemental Information 4 [file peerj-09-12523-s004.zip › Skull_VOI/DHMECN_SC_058_rec_Tra0607.png]

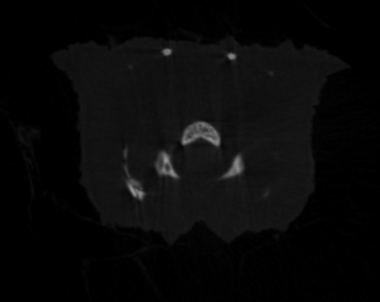

Supplement: Supplemental Information 4 [file peerj-09-12523-s004.zip › Skull_VOI/DHMECN_SC_058_rec_Tra0608.png]

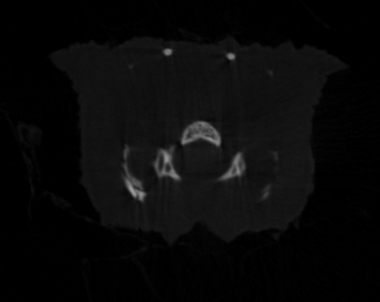

Supplement: Supplemental Information 4 [file peerj-09-12523-s004.zip › Skull_VOI/DHMECN_SC_058_rec_Tra0609.png]

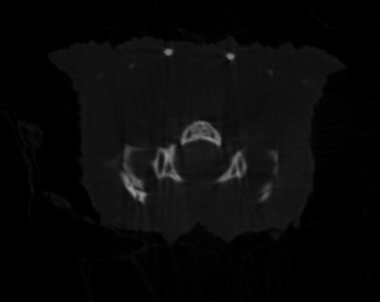

Supplement: Supplemental Information 4 [file peerj-09-12523-s004.zip › Skull_VOI/DHMECN_SC_058_rec_Tra0610.png]

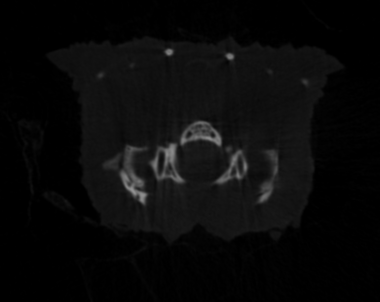

Supplement: Supplemental Information 4 [file peerj-09-12523-s004.zip › Skull_VOI/DHMECN_SC_058_rec_Tra0611.png]

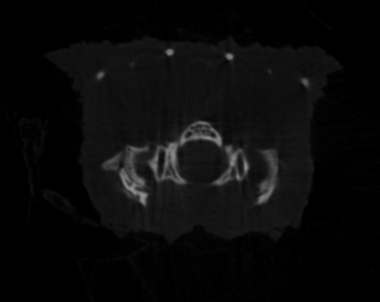

Supplement: Supplemental Information 4 [file peerj-09-12523-s004.zip › Skull_VOI/DHMECN_SC_058_rec_Tra0612.png]

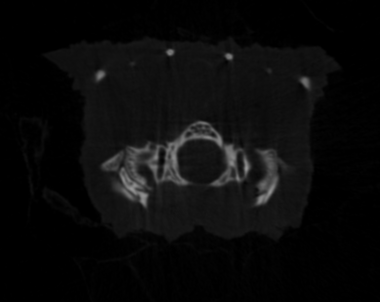

Supplement: Supplemental Information 4 [file peerj-09-12523-s004.zip › Skull_VOI/DHMECN_SC_058_rec_Tra0613.png]

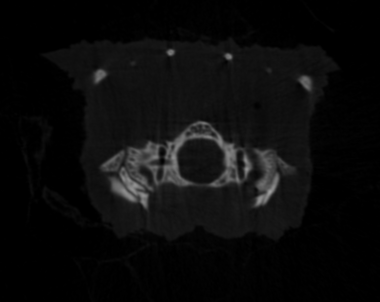

Supplement: Supplemental Information 4 [file peerj-09-12523-s004.zip › Skull_VOI/DHMECN_SC_058_rec_Tra0614.png]

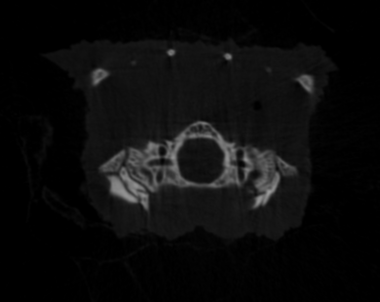

Supplement: Supplemental Information 4 [file peerj-09-12523-s004.zip › Skull_VOI/DHMECN_SC_058_rec_Tra0615.png]

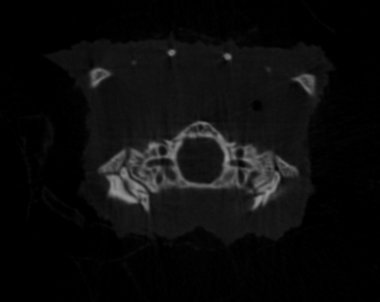

Supplement: Supplemental Information 4 [file peerj-09-12523-s004.zip › Skull_VOI/DHMECN_SC_058_rec_Tra0616.png]

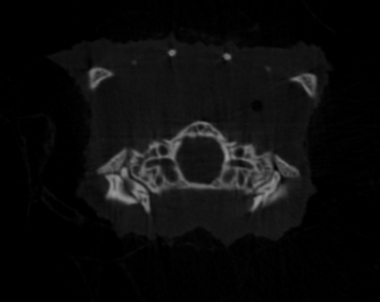

Supplement: Supplemental Information 4 [file peerj-09-12523-s004.zip › Skull_VOI/DHMECN_SC_058_rec_Tra0617.png]

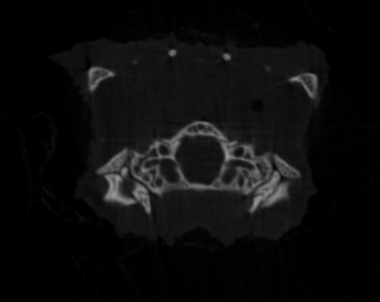

Supplement: Supplemental Information 4 [file peerj-09-12523-s004.zip › Skull_VOI/DHMECN_SC_058_rec_Tra0618.png]

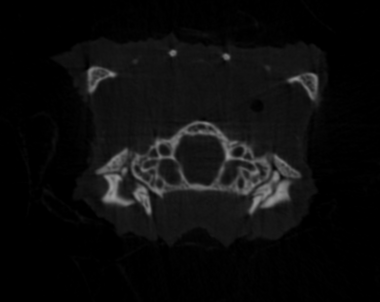

Supplement: Supplemental Information 4 [file peerj-09-12523-s004.zip › Skull_VOI/DHMECN_SC_058_rec_Tra0619.png]

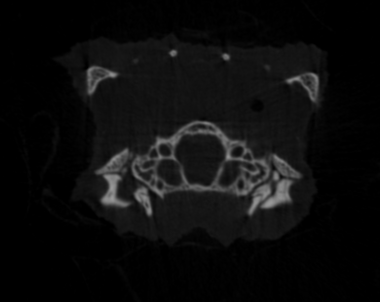

Supplement: Supplemental Information 4 [file peerj-09-12523-s004.zip › Skull_VOI/DHMECN_SC_058_rec_Tra0620.png]

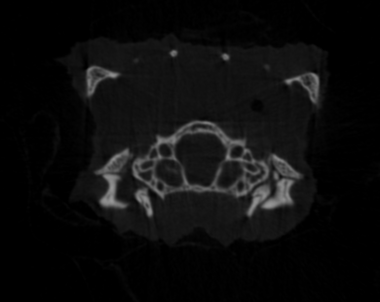

Supplement: Supplemental Information 4 [file peerj-09-12523-s004.zip › Skull_VOI/DHMECN_SC_058_rec_Tra0621.png]

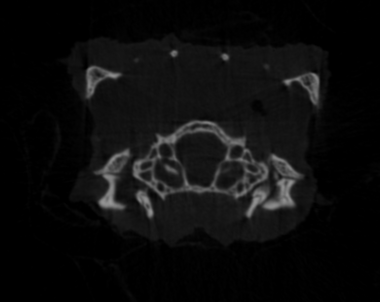

Supplement: Supplemental Information 4 [file peerj-09-12523-s004.zip › Skull_VOI/DHMECN_SC_058_rec_Tra0622.png]

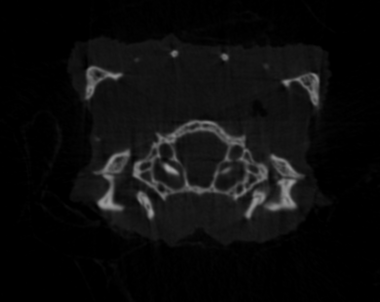

Supplement: Supplemental Information 4 [file peerj-09-12523-s004.zip › Skull_VOI/DHMECN_SC_058_rec_Tra0623.png]

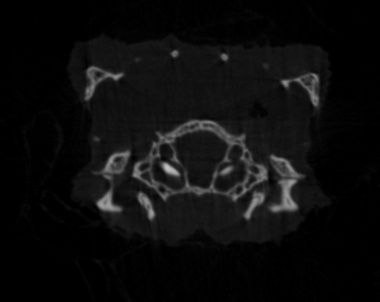

Supplement: Supplemental Information 4 [file peerj-09-12523-s004.zip › Skull_VOI/DHMECN_SC_058_rec_Tra0624.png]

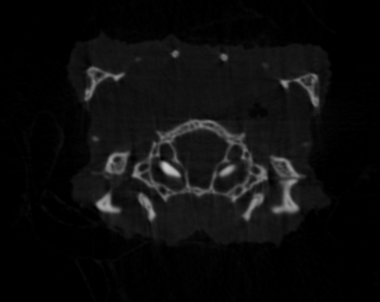

Supplement: Supplemental Information 4 [file peerj-09-12523-s004.zip › Skull_VOI/DHMECN_SC_058_rec_Tra0625.png]

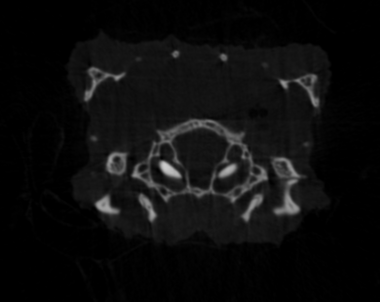

Supplement: Supplemental Information 4 [file peerj-09-12523-s004.zip › Skull_VOI/DHMECN_SC_058_rec_Tra0626.png]

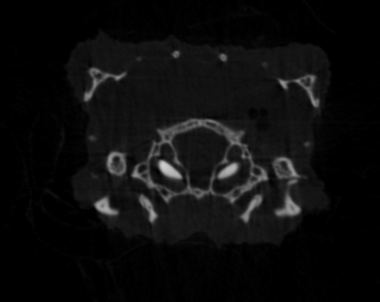

Supplement: Supplemental Information 4 [file peerj-09-12523-s004.zip › Skull_VOI/DHMECN_SC_058_rec_Tra0627.png]

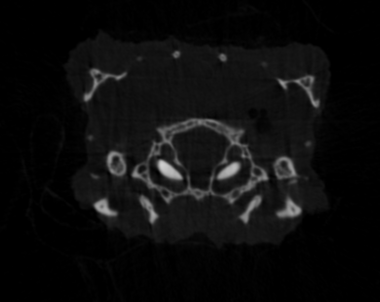

Supplement: Supplemental Information 4 [file peerj-09-12523-s004.zip › Skull_VOI/DHMECN_SC_058_rec_Tra0628.png]

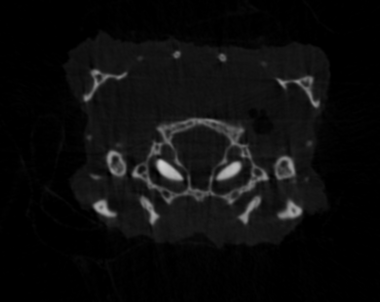

Supplement: Supplemental Information 4 [file peerj-09-12523-s004.zip › Skull_VOI/DHMECN_SC_058_rec_Tra0629.png]

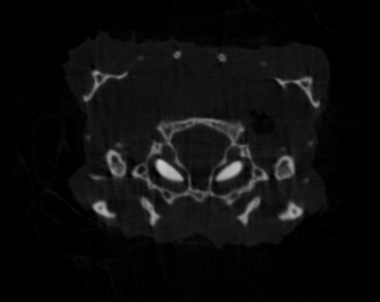

Supplement: Supplemental Information 4 [file peerj-09-12523-s004.zip › Skull_VOI/DHMECN_SC_058_rec_Tra0630.png]

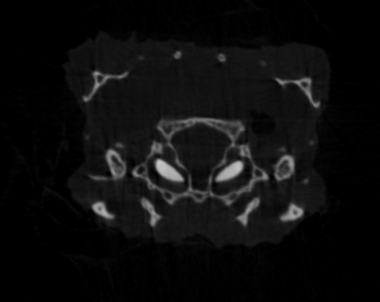

Supplement: Supplemental Information 4 [file peerj-09-12523-s004.zip › Skull_VOI/DHMECN_SC_058_rec_Tra0631.png]

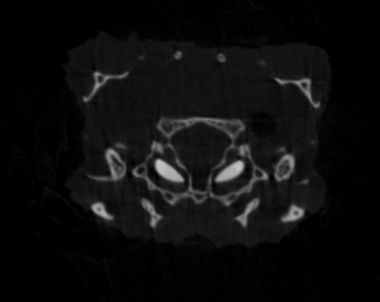

Supplement: Supplemental Information 4 [file peerj-09-12523-s004.zip › Skull_VOI/DHMECN_SC_058_rec_Tra0632.png]

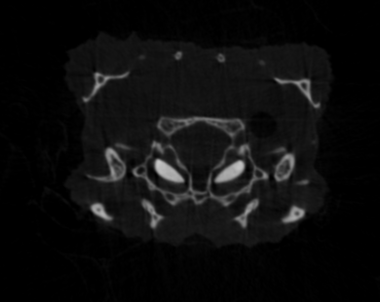

Supplement: Supplemental Information 4 [file peerj-09-12523-s004.zip › Skull_VOI/DHMECN_SC_058_rec_Tra0633.png]

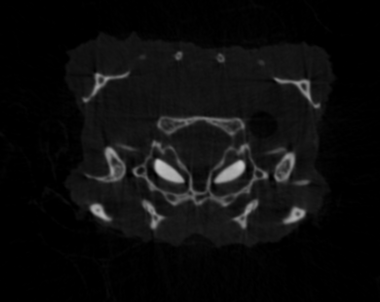

Supplement: Supplemental Information 4 [file peerj-09-12523-s004.zip › Skull_VOI/DHMECN_SC_058_rec_Tra0634.png]

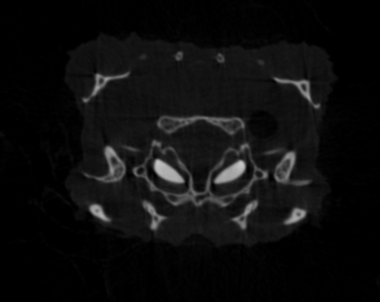

Supplement: Supplemental Information 4 [file peerj-09-12523-s004.zip › Skull_VOI/DHMECN_SC_058_rec_Tra0635.png]

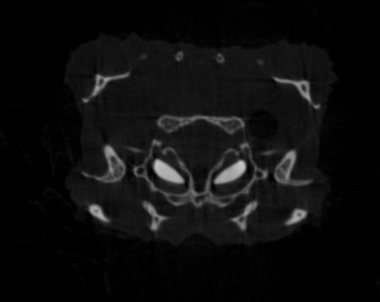

Supplement: Supplemental Information 4 [file peerj-09-12523-s004.zip › Skull_VOI/DHMECN_SC_058_rec_Tra0636.png]

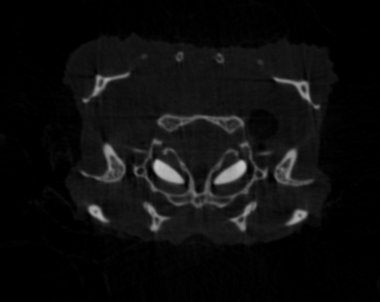

Supplement: Supplemental Information 4 [file peerj-09-12523-s004.zip › Skull_VOI/DHMECN_SC_058_rec_Tra0637.png]

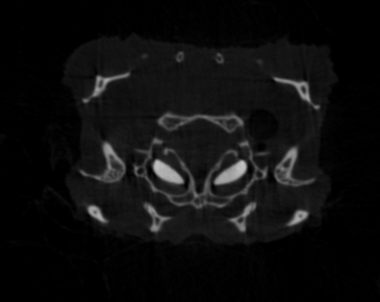

Supplement: Supplemental Information 4 [file peerj-09-12523-s004.zip › Skull_VOI/DHMECN_SC_058_rec_Tra0638.png]

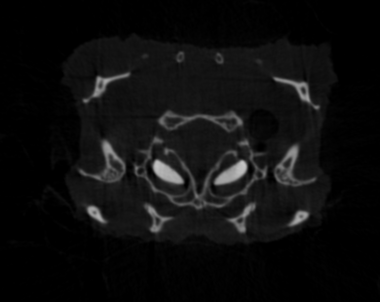

Supplement: Supplemental Information 4 [file peerj-09-12523-s004.zip › Skull_VOI/DHMECN_SC_058_rec_Tra0639.png]

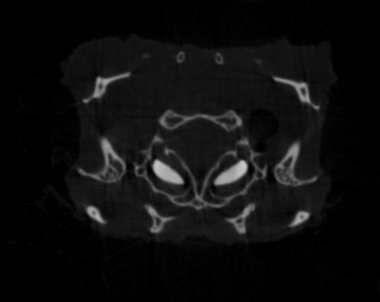

Supplement: Supplemental Information 4 [file peerj-09-12523-s004.zip › Skull_VOI/DHMECN_SC_058_rec_Tra0640.png]

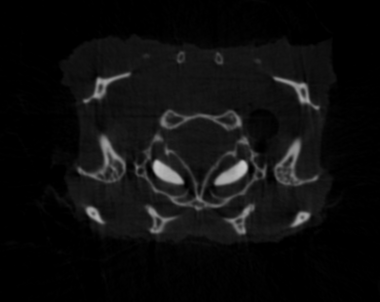

Supplement: Supplemental Information 4 [file peerj-09-12523-s004.zip › Skull_VOI/DHMECN_SC_058_rec_Tra0641.png]

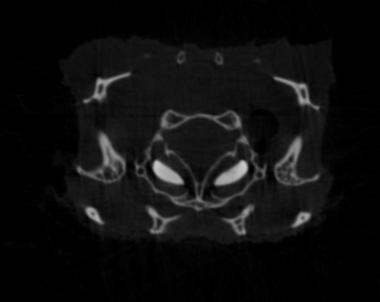

Supplement: Supplemental Information 4 [file peerj-09-12523-s004.zip › Skull_VOI/DHMECN_SC_058_rec_Tra0642.png]

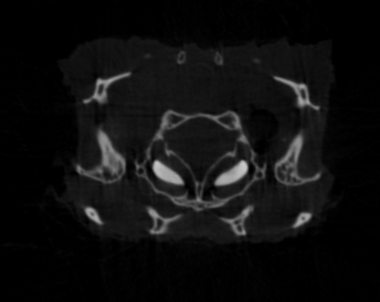

Supplement: Supplemental Information 4 [file peerj-09-12523-s004.zip › Skull_VOI/DHMECN_SC_058_rec_Tra0643.png]

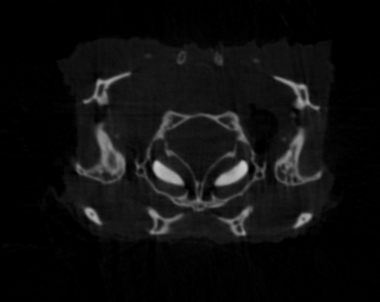

Supplement: Supplemental Information 4 [file peerj-09-12523-s004.zip › Skull_VOI/DHMECN_SC_058_rec_Tra0644.png]

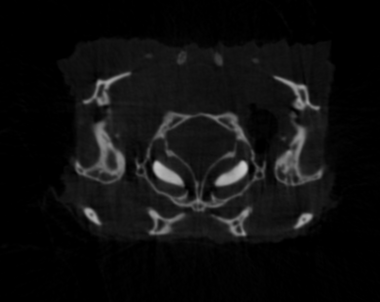

Supplement: Supplemental Information 4 [file peerj-09-12523-s004.zip › Skull_VOI/DHMECN_SC_058_rec_Tra0645.png]

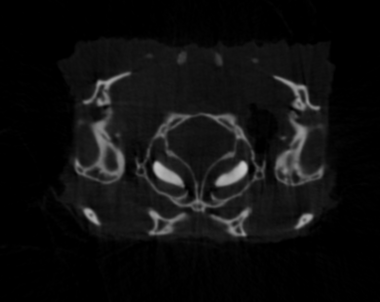

Supplement: Supplemental Information 4 [file peerj-09-12523-s004.zip › Skull_VOI/DHMECN_SC_058_rec_Tra0646.png]

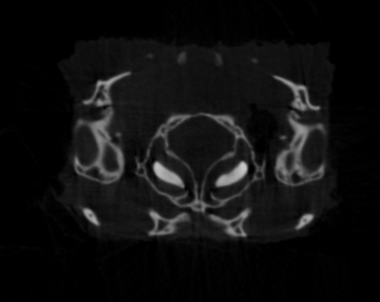

Supplement: Supplemental Information 4 [file peerj-09-12523-s004.zip › Skull_VOI/DHMECN_SC_058_rec_Tra0647.png]

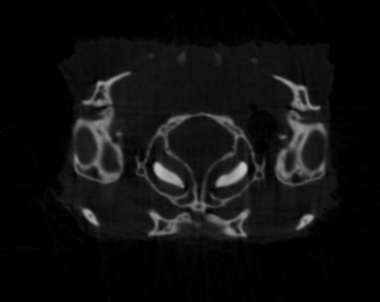

Supplement: Supplemental Information 4 [file peerj-09-12523-s004.zip › Skull_VOI/DHMECN_SC_058_rec_Tra0648.png]

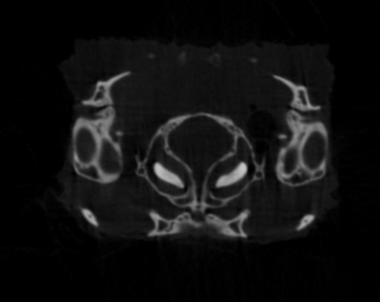

Supplement: Supplemental Information 4 [file peerj-09-12523-s004.zip › Skull_VOI/DHMECN_SC_058_rec_Tra0649.png]

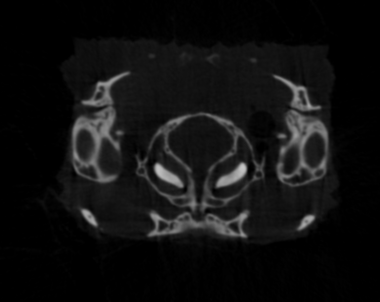

Supplement: Supplemental Information 4 [file peerj-09-12523-s004.zip › Skull_VOI/DHMECN_SC_058_rec_Tra0650.png]

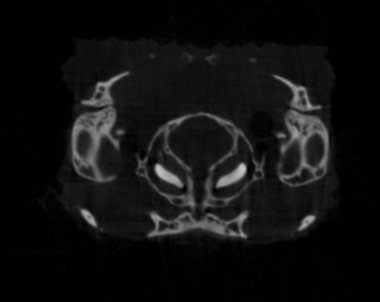

Supplement: Supplemental Information 4 [file peerj-09-12523-s004.zip › Skull_VOI/DHMECN_SC_058_rec_Tra0651.png]

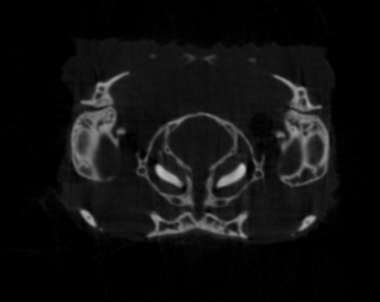

Supplement: Supplemental Information 4 [file peerj-09-12523-s004.zip › Skull_VOI/DHMECN_SC_058_rec_Tra0652.png]

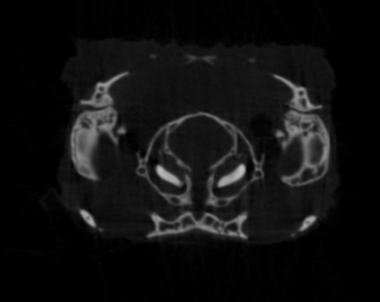

Supplement: Supplemental Information 4 [file peerj-09-12523-s004.zip › Skull_VOI/DHMECN_SC_058_rec_Tra0653.png]

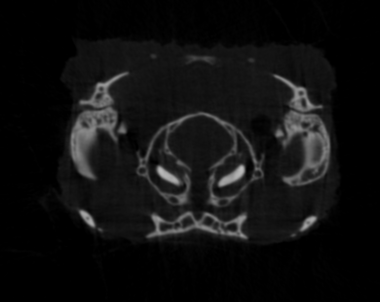

Supplement: Supplemental Information 4 [file peerj-09-12523-s004.zip › Skull_VOI/DHMECN_SC_058_rec_Tra0654.png]

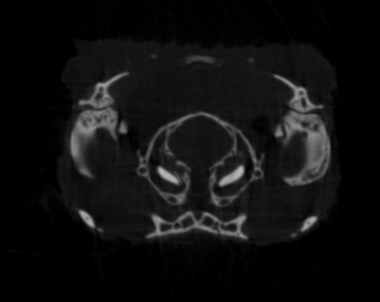

Supplement: Supplemental Information 4 [file peerj-09-12523-s004.zip › Skull_VOI/DHMECN_SC_058_rec_Tra0655.png]

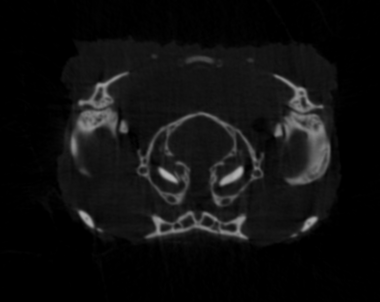

Supplement: Supplemental Information 4 [file peerj-09-12523-s004.zip › Skull_VOI/DHMECN_SC_058_rec_Tra0656.png]

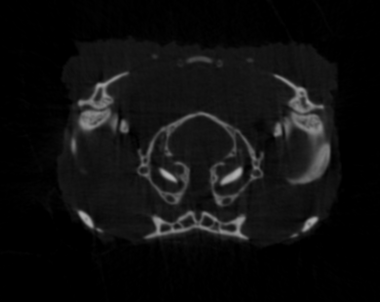

Supplement: Supplemental Information 4 [file peerj-09-12523-s004.zip › Skull_VOI/DHMECN_SC_058_rec_Tra0657.png]

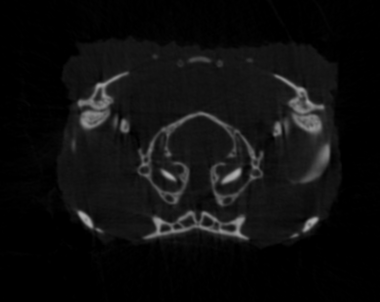

Supplement: Supplemental Information 4 [file peerj-09-12523-s004.zip › Skull_VOI/DHMECN_SC_058_rec_Tra0658.png]

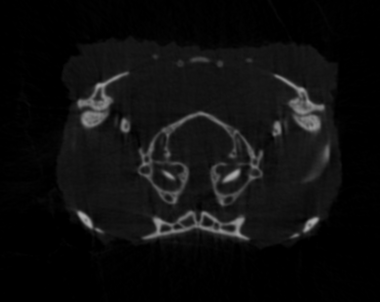

Supplement: Supplemental Information 4 [file peerj-09-12523-s004.zip › Skull_VOI/DHMECN_SC_058_rec_Tra0659.png]

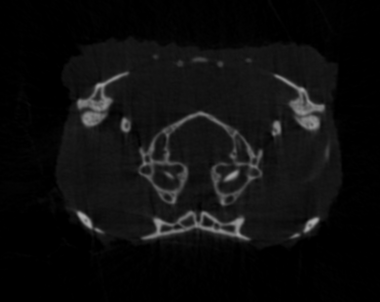

Supplement: Supplemental Information 4 [file peerj-09-12523-s004.zip › Skull_VOI/DHMECN_SC_058_rec_Tra0660.png]

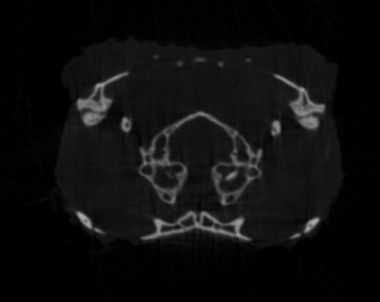

Supplement: Supplemental Information 4 [file peerj-09-12523-s004.zip › Skull_VOI/DHMECN_SC_058_rec_Tra0661.png]

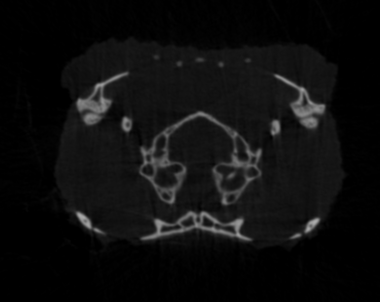

Supplement: Supplemental Information 4 [file peerj-09-12523-s004.zip › Skull_VOI/DHMECN_SC_058_rec_Tra0662.png]

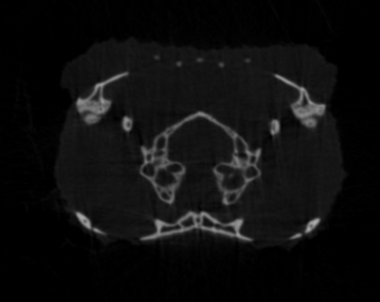

Supplement: Supplemental Information 4 [file peerj-09-12523-s004.zip › Skull_VOI/DHMECN_SC_058_rec_Tra0663.png]

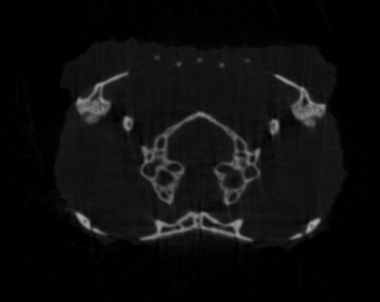

Supplement: Supplemental Information 4 [file peerj-09-12523-s004.zip › Skull_VOI/DHMECN_SC_058_rec_Tra0664.png]

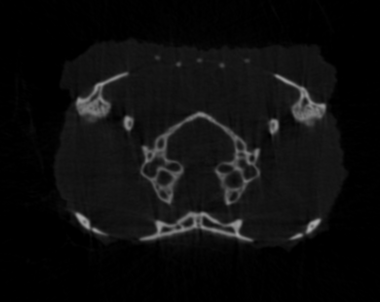

Supplement: Supplemental Information 4 [file peerj-09-12523-s004.zip › Skull_VOI/DHMECN_SC_058_rec_Tra0665.png]
